# Social influence and external feedback control in humans

**DOI:** 10.12688/f1000research.133295.2

**Published:** 2024-01-04

**Authors:** Martin Weiß, Mario Gollwitzer, Johannes Hewig

**Affiliations:** 1 University Hospital Würzburg, Würzburg, Germany; 2 Ludwig-Maximilians-Universität München, München, Germany; 3 Julius-Maximilians-Universität Würzburg, Würzburg, Germany

**Keywords:** feedback control, social influence, social interaction

## Abstract

This article aims to unravel the dynamics of social influence by examining the processes that occur when one person is the target of another’s influence. We hypothesized that these processes are part of a feedback loop system in an individual. This loop involves the situation (input), a goal state (reference), a comparator, a selection mechanism, a feedback predictor, and an action (output). Each element can become the target of social influence, and different types of social influence can be classified and explained by how these elements are targeted. For instance, attempting to persuade another person with strong arguments targets the goal state of the affected individual, while obedience targets the selection mechanism, and violence targets the action. In summary, this article aims to categorize, order, and explain phenomena in social influence research using a feedback loop framework focusing on the influenced individual.

## Introduction

Individuals often employ a variety of tactics in everyday life to exert influence over others ( Aiello *et al.*, 2018; Higgins *et al.*, 2003). For example, people may offer incentives such as gifts to persuade colleagues to assist with a challenging task, use rewards to encourage children to behave appropriately or present compelling arguments to convince academic peers of the validity of their theories. These tactics have been thoroughly investigated, and social influence is one of social psychology’s most extensively investigated research domains ( Harkins *et al.*, 2017). The present article seeks to provide a fresh perspective on social influence by examining the potential goals of an *influencer* toward an influenced *target.* Specifically, social influence means producing a particular effect in another individual, whether it is a thought, emotion, decision, or behavior ( Moussaïd *et al.*, 2013). Our perspective will focus more on the effects of social influence on an individual’s behavioral control system rather than their perceptual system.

We build on cybernetic models of behavioral regulation, that is, models that are designed to understand or regulate dynamic processes in complex systems ( Leonard *et al.*, 2021), focusing on the fundamental elements that comprise these models. Cybernetic models illustrate a closed system that regulates itself using a feedback loop. Social influence can target elements in cybernetic models, representing different forms of social influence.

We propose a framework that identifies beneficial and detrimental manipulation tactics in everyday life, contributing to a broader understanding of social influence. While previous research has identified different strategies of social influence ( Bruins, 1999; Higgins *et al.*, 2003), our approach exceeds existing approaches by categorizing these strategies according to distinct entrance gates to the internal feedback loop. Thereby, the proposed framework helps to develop a precise description of where and how different kinds of social influence affect another person’s internal feedback loop.

Our framework helps establishing a theoretical connection between cybernetic control models and other feedback regulation models, such as reinforcement learning ( *i.e.*, learning the optimal behavior in an environment to obtain maximum reward through observations of how choices are influenced by past decisions and rewards; Sutton & Barto, 1998). By combining these models in the future, researchers might be able to identify interactions that enhance our understanding and prediction of social influence. For example, combining our proposed model with reinforcement learning can help determine which social influence mechanisms are advantageous and crucial in social interactions.

## Feedback loops and feedback control

A feedback loop is a unit of cybernetic control consisting of four elements in a particular organization: input, reference, comparator, and output ( Carver & Scheier, 2000b). Input is often described as the is-state, while reference can be framed as a goal or ought-state. The comparator is a core component that detects the distance to the desired outcome (is-ought discrepancy), and the output refers to the actual behavior. Two types of feedback loops can be differentiated: one that aims to reduce discrepancies between the status quo and a goal (approach) and another that aims to increase discrepancies toward a particular anti-goal (avoidance). Thus, any social influence on another individual will either act through an approach or avoidance feedback loop. The cybernetic feedback model has been associated with the self-regulation of behavior, attention, and affect ( Carver, 2004; Carver & Scheier, 1981, 2000a). Different approaches have shown that social influence is related to emotional processes ( Fischer *et al.*, 2003), visual attention ( Frick *et al.*, 2018), and self-regulation ( vanDellen & Hoyle, 2010), thereby providing a connection between social influence and feedback loop models.

We believe that the cybernetic model is applicable to social interactions and understanding the self-regulation of behavior, attention, and affect in an actor. Moreover, exploring these processes in a target being influenced by an influencer is beneficial to understand interpersonal communication, improve leadership effectiveness, or to develop effective behavior interventions. Our approach is a potential step towards establishing a stronger connection between research on cybernetic action control, general action control theories, and the social influence literature.

In social interactions, actions elicit responses from others, leading to the possibility of feedback control where the actions of two or more parties influence each other. Feedback control in social interactions refers to the repeated process by which a person’s activities elicit reactions, generating a dynamic loop that determines subsequent behavior. This system requires constant adjustments depending on feedback and determines the continuing dynamics of interpersonal interactions. Classical frameworks have already investigated the translation of cybernetic regulatory principles into social phenomena ( von Bertalanffy, 1950; Wiener, 1949), providing insight into complex behavioral patterns, such as economic exchange and political negotiation ( McClelland & Fararo, 2006; Robinson, 2007). Jäger *et al.* (2015) have illustrated how the cybernetic feedback model applies to negotiation situations, where self-regulation functions as a feedback loop for goal orientation and identifies central challenges in negotiation processes. To address these challenges, specific self-regulation strategies were developed based on the individual components of the cybernetic feedback model.

Similar control systems were involved in controlling the behavior of social interaction partners. Motor movements ( *e.g.,* waving one’s hand) can be equated with communicative signals to other people ( Wolpert *et al.*, 2003). The principles governing social behavior, such as communication that influences our social world, also apply to motor behavior that impacts our physical environment. In each interaction, the actor generates motor commands based on predictions regarding the target’s potential reaction to the commands ( *e.g.*, raising one’s hand to ask for the waiter). The perceived target behavior is then compared with the predicted reaction, closing the loop of social interaction. Additionally, an individual might respond to the same input with many different patterns. For example, raising a hand in a lecture might indicate a question or stretching owing to tiredness. The professor’s response will depend on their interpretation of this social cue, either asking the student for their question or ignoring the signal altogether. Therefore, the interpretation of social signals may be ambiguous, and there is often a delay between the action and the intended reaction. This feedback loop demands a different approach than interactions with non-human physical objects, which are less likely to deviate from predictable behavior to the same degree.

Depending on the influencers’ objective, they can adapt their social behavior to reduce the discrepancy between the current and desired states. Feedback control theories have previously been applied to explain influencers’ behavior ( Diel *et al.*, 2021; Mansell, 2020; Sadiq *et al.*, 2021). We aim to extend this approach by using feedback control theories to explain the target’s ( *i.e.,* affected person) behavior. Accordingly, we want to examine the effects of the change in the feedback loop processes from the target’s perspective. Specifically, we seek to understand how and why certain aspects in the target’s feedback loop, which might not be overt, can be effectively influenced to achieve a desired outcome.

## Social influence

Social influence research has a long tradition, with the concept frequently discussed regarding social power ( McDonald & Crandall, 2015; Raven, 1964; Turner, 1991). In its early stages, power was defined mathematically as the maximum possible force “Person A” could induce on “Person B,” divided by B’s maximum resistance ( Lewin, 1941). Later, “influence” was defined as a force that an agent uses to alter the target’s behavior, opinions, attitudes, goals, needs, and values ( French & Raven, 1959). In their framework, French and Raven (1959) primarily differentiated between various types of exerting power, which will not be detailed here. Like influence, social power has often been defined as the ability to control or influence another’s thoughts, behavior, or feelings in a meaningful way ( Fiske, 1993; Thibaut & Kelley, 1959; Vescio *et al.*, 2003). In this line, more recent research on social power indicates that power might consist of two subcomponents, namely personal control and influence over others ( *e.g.*, Lammers *et al.*, 2016; Van Dijke & Poppe, 2006). The terminologies of “power” and “influence” are sometimes used interchangeably. French and Raven (1959) attempted to resolve this by defining influence as “kinetic power, just as power is potential influence” (p. 152), thereby distinguishing between potential and actual demonstrations of power.

It is important to note that not all social influences involve power. This article focuses on influence, defined as “attempts to affect or change other people” ( Levi & Askay, 2015, p. 128). Building on previous models, Raven (1992) shifted the focus from power subtypes to interpersonal influence, where agents are rational decision-makers who weigh the costs and benefits of their attempts to influence targets. This approach acknowledges that targets’ internal processes may adapt to agents’ attempts to influence them.

## Combining feedback loops and social influence

From a feedback loop perspective, extending the potential locus of influence may allow for a fresh perspective on human social interaction. Hence, we aimed to differentiate between different forms of social influence using the feedback loop perspective of the target of influence. Within this framework, there are various ways in which an external social agent can target elements of the feedback loop system of the target to achieve a desired outcome.

In adapting the cybernetic feedback model to social influence, we have slightly modified these terms coined by Carver and Scheier (2000b) to better suit our purposes. We refer to the reference value as a **goal state** as it represents the desired outcome of a sequence of actions. In our case, the input is described as the present social **situation**. During social influence situations, individuals learn from feedback received from the situation and make predictions about their own success, which we refer to as **feedback prediction**. This process affects the **comparator/reference**, just like the goal state and situation. In between these stages, we have included **action selection** and **execution**, which operationalize the output function and achieve the desired goal, thereby modifying the classical feedback model for social influence. Our adaptation of the cybernetic feedback model for social influence is supported by previous research in social psychology, which has highlighted the importance of social norms, expectations, and feedback in shaping behavior ( Cialdini & Goldstein, 2004; McDonald & Crandall, 2015; Wood, 2000), thereby providing a basis for understanding the processes involved in social influence. Importantly, some examples of influence aim to target an approach feedback loop in the target, while others address an avoidance loop ( Carver & Scheier, 2000b). These two types of loops are conceptually linked to promotion and prevention focused on the target ( Higgins, 1998, 1999).

**Figure 1.**
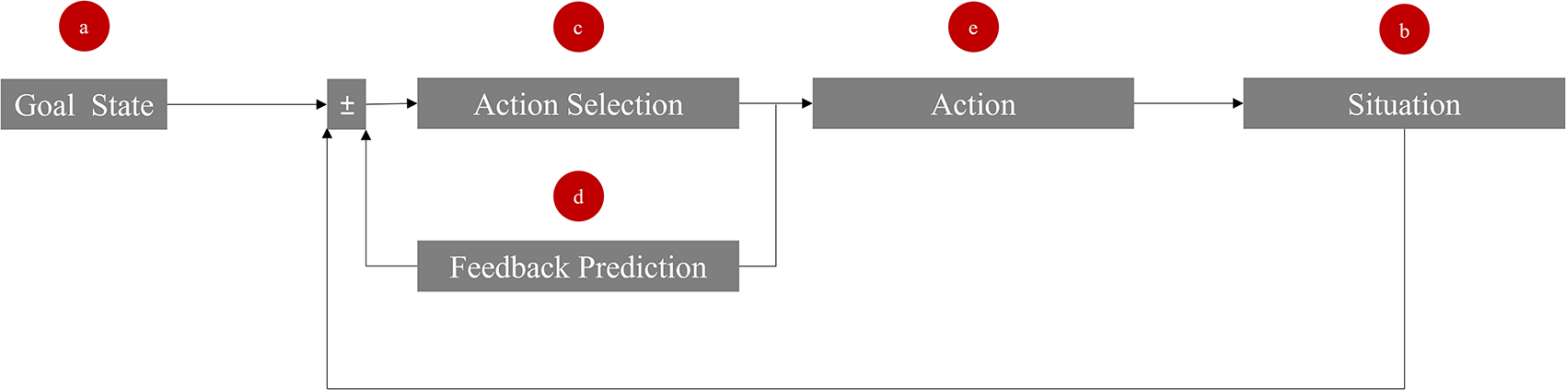
Five entrance gates (a-e) to manipulate the feedback loop in social interactions. The ± sign reflects the “comparator” and indicates that a possible discrepancy can be either positive or negative.

We propose a model comprising five key elements to access the internal feedback loop of a target, as illustrated in Figure 1. These elements are explained through the example of two roommates negotiating over the division of a shared television. The influencer uses the elements to manipulate the target’s internal goal state, situation, action selection, feedback prediction, and behavior.

Imagine two people—an *influencer* and a *target—*arguing about the division of a currently shared television. Let us consider a situation where the *influencer* wants to buy a brand-new television for him- or herself. The *influencer* may try to increase the value of the currently shared object and negotiate a deal to “sell” the shared television to the *target* to receive some money, by manipulating the *target*’s **goal state** (a), that is ‘owning the shared TV’ in this example. In this situation, the *influencer* persuades the *target* to understand that the *goal* ( *i.e.*, the shared television) is a worthwhile undertaking with advantages over other goal-like items. Alternatively, the *influencer* may want to keep the shared television and try to devalue it (the goal) to get the *target* to buy a new one. In this situation, the *influencer* might persuade *the target* that the *goal* is not worthwhile. Both strategies directly manipulate the *target*’s internal goal state or reference; in these examples, the *influencer* tries to amplify or attenuate the attractiveness of the *goal* for the *target* by either increasing or decreasing the value attributed to the *goal* and thus changing the desirability of the goal state. Hence, the amplification, attenuation, implementation, and elimination of a goal state may be relevant principal target strategies.

To manipulate the **situation** (b), the *influencer* can encourage the *target* to withdraw or to act. For example, the *influencer* could give the *target* a cheap TV as a gift to eliminate the discrepancy between not having a TV and wanting one, or remove the TV from the *target*’s access to increase the *target*’s desire for it. When the situation is manipulated, the comparator (which reflects the difference between the goal state, the current situation, and feedback prediction) can guide the *target*’s action selection.

When there is a misalignment between the *target*’s goal and their current situation, **action selection** (c) becomes important. The *influencer* can advise the *target* to encourage or discourage certain actions. For example, if *influencers* want to support the *target*’s goal pursuit, they can emphasize how difficult it would be to find a new one instead. On the other hand, if *influencers* want to discourage the *target* from achieving their goal, *influencers* can also suggest alternative actions to be selected, for example that the *target* buys a new TV. Depending on their interests, the *influencers* may suggest actions that increase or decrease the likelihood of the *target* achieving a goal about which they compete, including suggesting dubious alternatives.

Influencing the *target*’s **feedback prediction** (d) mechanism might motivate or discourage them by enhancing or lessening their expectations of achieving the goal. For example, the *influencer* could say, “I will make it very difficult for you to get this TV!” or “Why bother buying a new TV when you can have this one easily?”. Hence, the action might be encouraged or hindered by manipulating expectations; in particular, lowering the *target*’s expectations of success might result in frustration and disappointment, leading them to discontinue potential further efforts. Accordingly, feedback prediction mainly refers to the likelihood of reaching the desired goal state when selecting a certain action.

Lastly, during the **action** phase (e), the *influencer* can directly change the *target*’s behavior by making it easier or harder to perform certain actions. For instance, *influencers* might see the *target* packing up the TV and then remove it themselves from the house before the target can complete the action. In some cases, violent or deceptive methods may be used, such as physically stopping someone from running away, not paying a previously negotiated price because no contract was signed, or “accidentally” breaking the TV.

To illustrate our theoretical views and contribution to the literature, we provide specific examples of negotiation, social norms and sanctions, compliance, advertisement, and nudging in the following subsections. Thereby, we show how our feedback framework can be used to distinguish and understand the different forms of social influence. Prior to an in-depth exploration of each component within our proposed framework in detail, we want to clarify that our discussion emphasizes the predominant influence on individual components. This focus does not mean that influencing one component has no effect on other components. For instance, influencing the *target*’s action selection could also affect the goal state or situation. This interplay among components underscores the complexity of social influence, suggesting that interventions on one component may cascade into multifaceted consequences. However, our primary goal is to delve into the fundamental elements of influencing another person’ internal feedback loop. Developing an understanding of how to influence only one particular aspect while excluding the influence of another aspect can be crucial for a more detailed perspective on social influence.

## Social influence towards the goal state

To clarify how influence works, we outline the conceptual differences between persuasion and manipulation (see, *e.g.*, Gass & Seiter, 2022); then, we explore how changes in the *target* can be achieved through persuasion and how this relates to the *target*’s goal state. A differentiation between persuasion and manipulation is necessary since persuaders usually have transparent intentions and are open about their goals, whereas manipulators may hide their true intentions, using tactics that intentionally mislead or deceive others. Yet, both kinds of influence may be directed towards the target’s goal state.

Influence through persuasion can affect the goal state of the *target* even before the target takes any action. For influence directed toward achieving a particular goal, especially through communication and reasoning, the term “persuasion” is the most fitting descriptor. We do not aim at conceptualizing persuasion is a sub-facet of influence; rather, we use persuasion as a specific type of influence with a focus on communication and achieving a defined goal state. Falk and Scholz (2018) defined persuasion as changes in the preferences or behavior of recipients of information that conform to the active attempts of a communicator to promote such changes. According to the elaboration likelihood model (ELM; Petty & Cacioppo, 1986), a dualistic process accounts for attitude changes, which is relevant to changing goal states. The central route comprises a deliberate argumentation that requires a high degree of message elaboration to elicit a change in one’s attitudes and beliefs. Message elaboration refers to the extent to which individuals actively process information contained in a message. Thus, it involves the cognitive effort and depth of thinking invested in understanding and evaluating a message. In contrast, when a person lacks the ability or motivation to process a message in-depth, they may rely on the peripheral route, which means that peripheral cues or superficial aspects of the message rather than engaging in elaborate cognitive processing. These peripheral cues could be the influencer’s attractiveness or level of knowledge, which could lead to increased credibility. Taken together, we argue that persuasion as conceptualized here changes attitudes which in consequence will affect the choice of goal states of a target.

In addition to the influenced target route, the self-regulatory focus of the target is critical to determining the most effective manner of persuasion ( Aaker & Lee, 2001). Individuals with an independent self-view ( *i.e.,* a self-regulatory focus on promotion) prefer a persuasion focusing on approach motivation ( Aaker & Lee, 2001). In contrast, those with an interdependent self-view ( *i.e.,* a self-regulatory focus on prevention) prefer a persuasion that focuses on avoidance ( Aaker & Lee, 2001). Matching the persuasion/benefit of the interaction partner with the self-regulatory focus of the target leads to more effective persuasion ( Pentina & Taylor, 2013).

Aaker and Lee (2001) demonstrated these propositions through experiments, calling this mechanism a “central processing route to persuasion.” Although the authors did not apply a cybernetic view to their study, this perspective might be quite close to persuasion research. Their persuasive approach is characterized by its impact on the goal state or reference value, which constitutes the central mechanism in a cybernetic feedback model. Therefore, the persuasive approach aligns with the mechanism proposed by the cybernetic feedback model. Approach and avoidance motivation implicate the concepts of approach and avoidance goals ( *e.g.*, Carver and Scheier, 2000b), which have conceptual overlap with promotion and prevention focus, respectively. The necessity to match these to personal preferences of the target are well in line with the idea that this kind of persuasion is directed towards target’s goal states.

Related empirical evidence indicates that negative framing of information in a prevention-focused context is more effective in persuading individuals than positive framing, and *vice versa* for promotion-focused contexts ( Holler *et al.*, 2008; Spiegel *et al.*, 2004). For instance, Holler and colleagues provided a negative framing of insufficient tax provision, stating that “the health care system could not be maintained, and in case of illness outdated methods would be used” ( Holler *et al.*, 2008 p. 7). They found that individuals with a prevention focus were more tax compliant when presented with information emphasizing potential danger, and *vice versa.* Our framework aligns with the idea that persuasion is most effective when it matches the goal category, hence promotion or prevention domains.

Our proposed model suggests that persuasion can be most effective when transforming goals into action plans. This occurs when the goal is roughly defined, and the person considers the steps needed to achieve it. Manipulating goals can involve implementing a new goal or modifying an existing one, such as amplifying, attenuating, or eliminating it. To illustrate these differences, we additionally give the following examples.

Asch (1940) argued that the primary process in influence is not to change the attitude toward but the meaning of the object. Thus, changing the object’s meaning can lead to changes in implicit valuation, cognitive goal structures, attitudes, and behavior. Persuading individuals to frame desired goals can be an effective strategy for influencing subsequent action selection. However, framing requires the individual to have relevant knowledge about the object ( Nelson *et al.*, 1997). Framing can amplify or attenuate existing goals and their evaluation toward a specific direction using existing knowledge structures and potentially related long-term goals. Examples of this include emphasizing the emotional value of an object ( *e.g.*, highlighting the potential for promoting social gatherings like watching sports events with friends or romantic movies with a partner) or related long-term goals ( *e.g.,* emphasizing the potential of saving money if the target keeps the TV compared with buying a new one).

Haddock *et al.* (2008) discovered that matching persuasive messages’ cognitive or affective content with an individual’s initial attitude has a greater impact on goal persuasion than mismatched messages. Cognitive-oriented individuals are more susceptible to cognitive appeals, while affect-oriented individuals are more likely to be persuaded by affective appeals ( Petty *et al.*, 2009). For promotion-focused individuals, gain-framed messages are more persuasive, while loss-framed messages are more effective for prevention-focused individuals ( Cesario & Higgins, 2008; Lee & Aaker, 2004). Matching the message frame with regulatory focus results in greater fluency and influence ( Cesario & Higgins, 2008; Lee & Aaker, 2004).

Evidence from consumer research suggests that the way goals are framed affects persuasion significantly ( Min *et al.*, 2013; Poels & Dewitte, 2008). For example, framing goals as pursuing intrinsic rather than extrinsic objectives can lead to more desirable outcomes and higher engagement ( Vansteenkiste *et al.*, 2004). Therefore, framing intrinsic motivation, promotion focus, or matching initial attitudes can provide an effective surface for persuasion. This can be applied to the *influencer* in our example who tries to convince the *target* that watching TV does not align with their internal life goals ( *e.g.*, watching TV is a waste of time).

In persuasion, the fit between self-regulatory orientations (prevention and promotion) and comparative valence is crucial as persuasion is most effective when promotion-focused individuals are influenced by a positive comparative valence; the opposite is true of prevention-focused individuals ( Chang & Chou, 2008). This means that if the *influencer* aims to convince the *target* that the goal is unattractive, it will work best if the *target* is prevention-focused. Conversely, if the *influencer* wants to persuade the *target* to believe the goal is worth pursuing, this would work best if the *target* was promotion-focused. The influencer can attempt to amplify or attenuate a goal state within the target by downplaying or exaggerating its value, respectively.

A manipulator can influence goals to trigger conformity ( *e.g.*, the social goal to give group-conform responses), as shown by an experiment in which people estimated the movement of a dot of light ( Sherif, 1936). The individual assessments differed when people were alone versus in a group, suggesting that external factors can influence internal regulation. Social context can implement or amplify social goals in the manipulated person, such as maintaining social relationships or gaining recognition. Social influence is based on normative and informational influence ( Deutsch & Gerard, 1955; Legros & Cislaghi, 2020); normative influence aims to conform to expectations, while informational influence aims to reduce uncertainty. Influencers can address both types of conformity and even influence internal goals. Conformity can regulate persuasive processes and affect other levels of the feedback loop, such as restricting or postponing the selection of actions.

## Social influence on the subjective situation

To impact a situation, individuals need to be aware of the situation and how to influence it. In our shared television example, the *influencer*’s belief in their ability to win the argument can affect the outcome. For example, if the *influencer* is the property owner, they could change the situation by declaring the TV as part of the apartment inventory, altering the legality of the negotiation. This action could make the *target* rethink their financial situation and potentially withdraw from the negotiation, reducing the risk of a physical conflict ( von Hippel & Trivers, 2011).

Another example of how influence affects the situation is that people are likelier to litter in a polluted environment than in a clean one ( Cialdini *et al.*, 1990). However, confederate littering in both environments had contrasting effects: it led to an increase in littering in the polluted environment but a decrease in the clean one. This indicates that situational cues can influence behavior. In the roommate example, as suggested above the influencer could give the *target* a cheap TV as a gift, or remove the TV from the *target*’s access and thus changing the situational context. Alternatively, the *influencer* could manipulate the negotiation situation by choosing a location that gives them an advantage. Let us say the *influencer* is good at gambling at which the *target* is not good and makes the *goal* part of a stake, compromising the *target*, who might feel uncomfortable in the desired scenario. Such an influence changes the current and subjective situation of the target, resulting in subsequent consequences in the feedback loop. The discrepancy between the goal state and the situation ( *i.e.,* the *goal* is not yet in the target’s possession) becomes amplified. The reason for this amplification is not the absolute difference between the goal and situation, as the *goal* remains unchanged and is still not in the *target*’s possession; instead, the *target* may be less capable of demonstrating behavior that could successfully lead to the goal. This ultimately means that the feedback prediction or success probability is lowered. Consequently, a larger discrepancy is created in the comparator.

Social comparison is an adjacent concept that alters the perception of a situation instead of the facts. Social comparison involves comparing oneself to others, which can influence how individuals perceive their own circumstances. Hence, the influencer’s actions may stimulate the target making either upward or downward social comparisons. Upward comparisons aim to increase achievement, while downward comparisons aim to increase subjective well-being ( Diel *et al.*, 2021; Wheeler, 1966; Wills, 1981). Accordingly, the *target* may perceive the situation differently when making either of these two kinds of comparisons. In influencing the television negotiation, the *influencer* might point to newer and better TV models if the intention is to keep the shared TV; if the intention is to sell it, the reference may be to older TV models or the benefits of the shared TV.

The specific reason for influencing a situation is often aimed at subsequently affecting the different mechanisms of the cybernetic feedback model. According to the mechanism of upward and downward comparison, an influence on the perceived state may provoke a discrepancy in the comparator of the feedback loop or obscure an existing one. Furthermore, it is possible to influence the situation to increase the basic physical distance between the goal and the target. Another reason for influencing might be to decrease action opportunities and, thus, influence action selection. In both cases, the performance of a successful action by the *target* becomes increasingly difficult, indicating that influence decreases the probability of success, affecting feedback prediction.

## Social influence on action selection

Persuasion can alter the internal feedback loop of others during action selection. While the concept of persuasion is widely studied in social psychology, we will only discuss a relevant aspect of persuasion in our article—our interpretation of persuasion based on the feedback framework and its impact on the feedback loop. It is difficult to isolate persuasion solely aimed at action selection versus goal structures, such as an individual’s basic preferences. However, differentiating between the two types of persuasion could aid in understanding the persuasion mechanism. We aim to provide a systematic persuasion approach based on the feedback loop’s affected aspects. In terms of the direct influence on action selection influencers may promote and prevent certain actions of the target by advising and coaching the target and by directly suggesting to select a specific action as compared to another one.

When doing this, matching the argument in the *influencer*’s statement to the function underlying the *target*’s attitudes is an important aspect of persuasion ( Lavine & Snyder, 1996). People have different attitude functions for various issues, and not all are equally receptive to persuasion. Knowing the *target*’s attitude functions helps the *influencer* evaluate the effectiveness of their persuasive attempts, and using convincing arguments instead of weak ones is crucial for successful persuasion ( Petty & Wegener, 1998) when suggesting the target to favor one action option as compared to others. Persuasive messages targeting important attitude functions are processed carefully and are less influential ( Marsh *et al.*, 1997). Several factors contribute to the success or failure of persuasive attempts aimed at action selection and influencing the internal feedback loop of the *target.*

Chambon and Haggard (2012) proposed a model on the prospect aspect of agency, suggesting that early signals reflecting action selection contribute to a sense of control. A positive sense of agency helps individuals adjust to a dynamic environment and direct their behavior toward a goal ( Elsner & Hommel, 2001; Hommel & Elsner, 2009; Ren *et al.*, 2023). Thus, it is useful to reinforce prioritized actions that lead to control over the environment ( Redgrave *et al.*, 2008; Redgrave & Gurney, 2006). It is challenging to influence directly at this stage. However, an external source might be able to manipulate the internal feedback loop; arguments previously expressed by others before action selection may come to mind at this stage. Accordingly, arguments in favor of an action option should promote the prospect of agency and an expected high sense of control.

In contrast, to make some action options less likely, the opposite would be promising, namely a reduction in the expected agency and sense of control. In the context of the shared TV example, imagine that the *influencer* wants to donate the TV to charity, but the *target’s* goal state would be to keep it. If the *influencer* posts about giving it to charity on social media, knowing the *target* will see it, it could lead to the *target* withdrawing their own action plan to get the TV since they might feel unable to oppose such a socially desirable action option. In this case, the *influencer* would have promoted an alternative course of action by raising doubts in the *target* about their own potential action. The *action selection* in the *target* may thus result in withdrawing from keeping the TV and the decision to let it go. This influence strategy is based on the *target*’s re-evaluation processes and could be successful for the *influencer.*

Another way to influence action selection is through obedience ( Caspar *et al.*, 2021). Milgram’s (1963) experiments demonstrated how people comply with commands despite potentially harming others. In these experiments, a fictional authority-subordinate relationship was established to create a goal state that required a predetermined action ( *i.e.,* administering electrical shocks to an unseen receiver). Later research replicated these findings, showing the importance of predetermined action selection as a potential influence strategy ( Slater *et al.*, 2006). The use of electric shocks as a means of social influence highlights the complexity of social influence and the need for a nuanced understanding of the underlying processes. For instance, the experiment could also influence the goal state, as using electric shocks for education may differ depending on one’s moral objections.

According to Stayton *et al.* (1971), humans are receptive to social influence through external signals rather than internalized control. This suggests that humans are prepared to obey verbal instructions. The foot-in-the-door technique ( Freedman & Fraser, 1966) is an example of compliance without the involvement of an authority figure. This technique initially makes a small request, followed by a larger related request. The strategy is successful where the compliance rate is higher for those who received the original request than those who did not ( Freedman & Fraser, 1966).

The influence of an *influencer* on the *target*’s action selection can be affected by various factors, such as the *influencer*’s perceived reputation and trustworthiness. If the *target* perceives the *influencer* as highly reputable and trustworthy ( Haslam *et al.*, 2014; Hollander & Turowetz, 2017), they may be more likely to obey or comply with the *influencer*’s advice. This is true, even if the advice does not necessarily align with the *target*’s intuitive approach to achieving their goals.

In our shared TV example, the *influencer* might suggest a specific action that is purposeful but may not align with the *target*’s initial preferences. However, this tactic is more likely to be effective if the *influencer* is perceived as highly reputable and trustworthy, which may lead the *target* to comply. Depending on the *influencer*’s goal, they may suggest actions that increase or decrease the probability of the *target* achieving their goals. For instance, an *influencer* may suggest dubious action alternatives to compromise the *target*’s success rate or portray an authority that the *target* should obey, especially if the *influencer* appears to have more experience or technical knowledge.

Other social manipulation tactics, such as coercion, hardball, or silent treatment (see Buss, 1992), also fall under this category. These tactics use strong aversive motivational tools to push a particular action selection in the *target.* However, this article does not address these tactics in more detail. Another potential approach to influence action selection is nudging, which uses positive reinforcement and indirect suggestions to encourage a particular behavior or decision without restricting the individual’s freedom of choice ( Sunstein, 2014).

In summary, the effectiveness of an *influencer*’s strategy in influencing the *target*’s action selection depends on various factors, including the *influencer*’s perceived reputation and trustworthiness, their interests, and the type of approach they use.

## Social influence on behavior while acting

The influence on actual behaviors is particularly challenging to isolate in the proposed cybernetic feedback model. In this article, we tried to address attentional, perceptive, or cognitive manipulations in the stages preceding entering a feedback loop. Hence, we refer to physically-induced manipulation when discussing behavior in this subsection. This subsection comprises influences closely linked to behavioral change, such as violent or deceptive action redirections ( *e.g.,* pretending to be a courier for someone before delivering completely different information).

However, most social influence research concerns social norms and subtle psychological processes ( Cialdini & Goldstein, 2004). Thereby, descriptive norms refer to the behavior of relevant others ( *e.g.*, peers, family, society) and provide a socially determined standard for effective behavior ( *i.e.*, what individuals typically do), while injunctive norms refer to appeals about what other members of society approve or disapprove of. Descriptive norms, for instance, can be sufficient to alter an individual’s behavior through exposure to peer groups ( Cullum *et al.*, 2010; Goldstein *et al.*, 2008). The social influence induced by social norms can alter the internal feedback loop regarding action selection but also action itself. Specifically, exposure to peer groups’ behavior can prime and make a selected action more salient ( Do *et al.*, 2022) or the mere adaption of an action more likely ( Albert *et al.*, 2013). In contrast, the ability to deduce intentions from peers’ behavior can lead to the formation of goals (for a review on peer influence in energy consumption behavior, see Wolske *et al.*, 2020). Another interesting aspect in this context, which is not discussed in detail here, is the identity-based group influence as discussed in a recent review ( Spears, 2021).

In addition, actively promoting and enforcing social norms can alter the future behavior of others. For example, people may ostracize norm-deviant behavior to strengthen their position in a group ( Willer *et al.*, 2009). This directly affects the punished person and indirectly influences others through previously mentioned mechanisms. The punishment may implement an avoidance goal, influence action selection by favoring active avoidance choices and alter feedback prediction by reducing the expected success of behavior opposing the influencer.

The distinction between physically-induced manipulations and social influence research is important in understanding the various mechanisms of social influence. While physically-induced manipulations are the focus of this opinion piece in the current section, social norms and influence processes are important to altering related feedback loop behavior. By understanding these mechanisms, we can gain insight into how behavior can be altered in feedback loops, both directly and indirectly.

## Social influence on feedback prediction

In reinforcement learning theories, feedback prediction is the predicted reward value of performing an action. Our brains utilize these values to adapt behavior by biasing action selection towards instrumental actions with the highest predicted reward value ( Redgrave *et al.*, 1999; Sosa & Giocomo, 2021). In the broader sense of value based on expectancy models, feedback prediction optimizes goal pursuit and combines reward value and the probability of success. Those behavioral actions that are instrumental to achieving goals with higher predicted outcome values are chosen more often ( Samejima *et al.*, 2005) and more rapidly ( Brown & Bowman, 1995) than those with lower values. Furthermore, the reward system is also known to prefer actions and goals that implicate actual ( Bednark & Franz, 2014; Behne *et al.*, 2008) versus potential ( Leotti & Delgado, 2011; Tricomi *et al.*, 2004) control over the environment.

In our proposed model, the *target’s* internal feedback loop can be altered by manipulating their subjective probability of success; it can be increased to stimulate or motivate action or reduced to discourage it. As mentioned (see “social influence on the subjective situation”), manipulating the situation may often be a valid pathway to influence feedback prediction.

In some cases, the *influencer* may use deception to instill a false belief in the *target*’s mind to reach their goal. We use deception as a functional strategy to achieve goals in a social interaction by influencing others in their feedback prediction process. The central aim is to alter the *target*’s success predictions, either by exaggerating them to promote success or abridging them to discourage it. We will not delve into the consequences of falsehoods or affective aspects that may arise when deception is detected, as we focus on the framework for entering a feedback loop.

Framing is key in achieving this type of influence, as it emphasizes the distinct attributes of an issue over other potential consequences ( Nelson *et al.*, 1997; Nelson & Kinder, 1996; Price & Tewksbury, 1997); its effectiveness seems to depend on familiarity with the issue. The reason might be that persons familiar with a certain topic have a deeper knowledge structure than individuals unfamiliar with it. In the context of our shared TV example, a devaluation framing could be a phrase like “I will make it very difficult for you to get this TV!” or the opposite might be a phase like “Why bother buying a new TV when you can have this one easily?”. Many studies have explored this persuasive strategy ( Druckman, 2001; Gächter *et al.*, 2009; Tversky & Kahneman, 1981) though framing may sometimes also address the valuation of the goal state and would then be related to the respective section above.

## Examples of model applications

In the following subsections, we describe studies dealing with different aspects of social influence and persuasion. We outline how our model differs characteristically and alters the internal feedback loop of participants at different stages. In Higgins’ (1997) regulatory focus theory, the authors posit two distinct self-regulatory orientations—prevention and promotion focus—relevant to our model as they directly influence the cybernetic feedback loop. Following that model, we posit that a prevention or promotion feedback loop will occur in a target person. There may be overlapping aspects between—but also within—the studies regarding the different entrance gates to manipulate the *target*’s feedback loop, which we will present in the following subsections. Moreover, not all aspects of our model will be addressed and easily implemented in every experiment. However, by altering the experiments, we propose simple and creative solutions to describe manipulation options according to our model. While the mechanisms of our cybernetic feedback model could most effectively be investigated through laboratory experiments, we have decided to include both field and laboratory experiments.

Before starting with the research examples, we will briefly describe the systematic approach used to analyze studies in this domain. First, we identified the relevant goals in the experiment ( *e.g.,* social goals, personal goals) and whether their value was manipulated or an additional goal was included. For instance, introducing a social comparison may add social goals to an otherwise private context. Second, we analyzed the influenced situational characteristics. Third, we examined whether action selection was manipulated—whether a specific behavioral choice was promoted. Fourth, we examined action opportunities or direct effects on action execution. Fifth, we examined the effects of social influence on feedback prediction and ‘goal state versus situation’ discrepancy, for example, by manipulating a *target’s* subjective goal attainment probability.

In the following subsections, we explain the findings, apply our model, and derive additional experimental manipulations based on our framework.

## Descriptive and injunctive social norms

Melnyk *et al.* (2013) argue that descriptive norms motivate engagement in specific behavior depending on their social rewards, while injunctive norms motivate engagement through the threat of punishment. They investigated the effect of regulatory focus on the influence of descriptive and injunctive social norms in the context of sustainable food choices. We use their study as an example of a potential influence on the goal state and (indirectly) action selection.

In the first three experiments by Melnyk *et al.* (2013), state regulatory focus was induced as a between-variable to participants. For norm induction, participants were shown a fictitious website covering information on fair-trade coffee using a text framed with either descriptive or injunctive norms. Participants were then asked to answer questions regarding attitudes toward fair-trade coffee and their planned buying intentions. While controlling for past buying behavior, an induced promotion focus increased the efficiency of descriptive norms, resulting in more favorable attitudes and intentions toward sustainable choices. In contrast, injunctive norms were not influenced by the regulatory focus state.

While the second experiment by Melnyk *et al.* (2013) only targeted the perceived fluency of the different normative messages, the third experiment replicated the first, using organic milk instead of fair-trade coffee. Here, regulatory focus induction was included in the text message and not conducted separately, as in the first experiment. However, the results were comparable to those of the first experiment. When taken together, the results showed that promotion focus increased buying intentions in the descriptive norm condition, while no differences were found in the injunctive norm condition. This may be due to a ceiling effect since intentions were as high in the injunctive conditions as in the promotion-focused descriptive conditions. The former aligns with descriptive norms’ influence on a promotion-feedback loop.

As participants in the studies conducted by Melnyk *et al.* (2013) were invited to evaluate the design of a website, their goal orientation was distracted from the actual research goal. However, social comparisons regarding relevant others ( *i.e.*, other students), which were used in the descriptive norm induction (“A great number of […] students purchase Fair Trade coffee regularly”), might have activated an explicit awareness of social norms regarding pro-environmental behavior. Importantly, environmental goals were not different between conditions, so only the presence of different social goals was relevant.

Particularly, the presence of an injunctive norm generally increased intentions, so it was sufficient to influence behavior. According to our model, we argued that this was due to the inclusion and presence of a social goal. In the descriptive norm condition, we suggest that the presence of a promotion focus was sufficient to activate a goal in participants. Moreover, participants in the studies of Melnyk *et al.* (2013) conducted the experiment together in one room, facilitating implicit social comparisons with peers. Consequently, the situation was influenced by the data collection ( *i.e.*, in groups), indicating that the state-induced regulatory focus might have become fragile, particularly for individuals sensitive to social pressure.

In the studies conducted by Melnyk *et al.* (2013), action selection was manipulated by the different normative framings present in the website content. However, as participants were instructed to focus on design features, the target manipulation tool of the study was implicit. The distraction from the study goal might have enabled participants to answer the dependent variable questions for reasons other than experimental manipulation ( *e.g.*, general pro-environmental attitudes). Nonetheless, the experiments did not directly influence action selection because they were based on text reading and questionnaires. However, the success of feedback prediction depends on interindividual differences. Individuals with pro-environmental attitudes and a low budget might have perceived the situation as a trigger for a discrepancy between the personal goal state and the is state ( *i.e.*, they would like to buy fair-trade coffee but cannot afford it). Regarding the apparent study goal—evaluating a website design—a discrepancy between the goal state and the situation might only have been present if the website design was overly flawless. Such a scenario would have caused participants to be unable to offer suggestions and fail to achieve the goal.

Future research based on the cited study might add several conditions and prerequisites to investigate the interaction between regulatory focus, descriptive norms, and the first and injunctive norms. We propose two additional conditions with the explicit goal of focusing on the text by asking participants whether the content could lead to more purchases of fair-trade products (versus a control group). Therefore, differences in the goal state and the influence on action selection could be examined using our framework. Furthermore, we recommend that future studies focus more on goal-framing than on peers’ behavior; for instance, buying fair-trade coffee could be presented to students as promotional material for local and sustainable food production or preventing the exploitation of local resources.

In two field experiments, Goldstein *et al.* (2008) investigated the influence of three different towel reuse signs in hotel rooms on pro-environmental behavior. The hotel guests were not informed about their participation in the experiment and were randomly assigned to one of three towel-reuse conditions. This example serves to highlight the influence on action selection beyond the goal state more clearly.

In the first experiment, the first condition corresponded to an industry standard that emphasized towel reuse for environmental reasons without referring to any descriptive and normative information. The second condition corresponded to a global standard describing the towel reuse behavior of all hotel guests. The third condition was a provincial norm describing the towel reuse behavior of other guests who had booked the same room. The results showed that the third condition (specific hotel room) significantly impacted behavior; the provincial norm led to more towel reuse than the global and industry standards. In the second experiment, the authors replicated the findings of the first experiment, showing that hotel guests conformed more to the provincial norm compared with the norms of other hotel guests ( *e.g.,* gender identity). ^1^ Unfortunately, there was no control condition for this experiment.

The major difference between the three main conditions was the presence of a social comparison in the second and third conditions. This may introduce additional social goals and social motivation for the target. Considering these additions, participants were prompted to compare their behavior with the proposed social comparison group. This may have motivated behavior through goals such as social attachment. As the hotel guests were ostensibly unaware of their study participation, they were also unaware of the manipulation of the goal state and the study aim. This study design has an advantage over laboratory experiments, although the latter might induce an artificial focus on social norms. However, environmental characteristics, such as the hotel type (in this case, a “midpriced chain hotel”), might have subconsciously implemented a general behavioral goal; for instance, an eco-hotel might increase pro-environmental behavior. Replicating these experiments using a controlled setting would allow for investigating general pro-environmental behavior without any norms ( *e.g.,* avoid energy-wasting behavior), which might more accurately represent an internal goal state than the signage would.

An alternative experimental manipulation of the situation could be to measure the number of towels reused per day compared with relative numbers; the situation, in this case, depends on the number of booked nights. With this approach, researchers may assess behavioral changes for guests with different bookings, identifying potential differences between staying only one night and staying one week, for example. In another hypothetical scenario, the signs might focus on different standards rather than specific reuse goals. For example, if the goal is to reuse 10 towels per week, participants’ behavior might differ if eight have already been reused when they enter the room compared with a situation where only two have been reused.

The selected study aimed to manipulate action selection using different signs. However, the most effective method for manipulating action selection was to address the current situation of participants ( *i.e.,* the towel reuse behavior of other guests who had booked the same room). This example illustrates that, during the physical absence of social agents, action selection needs to and can be manipulated *via* a preceding operation ( *i.e.,* manipulating the goal state or the situation). In this scenario, one possibility to influence action directly, in an ethical manner, would have been to implement a condition where cleaning staff would put the towels back, thereby forcing participants to reuse them. Participants would then have to invest more energy into not reusing the towels; for example, hotel guests may need to specifically request that staff clean the towels.

An additional reward or token system for towel reuse behavior could be applied to directly address the action and feedback prediction simultaneously. In a cybernetic feedback model, when there is a significant difference between an individual’s internal reference value (such as their preference for reusing towels) and external information (such as a feedback sign in a hotel bathroom evaluating their behavior), this can lead to a larger disparity in the comparator. As a result, the individual may make more significant adjustments in their action selection and execution to decrease this discrepancy, for instance, increasing towel reuse if their initial preference for reusing towels was low. By extending and altering the study setup with these examples, future studies could investigate which pathway for addressing social norms might best foster pro-environmental behavior within a feedback loop framework.

## Compliance

To illustrate the merit of our model for compliance, we can apply our framework—especially the influence on the goal state—to research related to health promotion and disease prevention in healthcare ( Spiegel *et al.*, 2004) in a study on the nutritional habits of college students. Participants received a daily nutrition log booklet to record their daily consumption of fruits and vegetables; they were instructed to return a week later with a completed booklet. Participants received a questionnaire on food habits and health messages along with the booklet. Using a between-subjects design, participants received four different messages framing the regulatory focus (addressing the imagined benefits of compliance) and the outcome (addressing the imagined costs of non-compliance). The dependent variable was the effectiveness of health messages in changing behavior: eating more fruits and vegetables after one week. Results revealed that messages that focused on the potential benefits of successful dietary change were more effective in promoting behavior change when the goal was presented as health promotion-focused, compared to messages that focused on the potential costs of dietary change failure. The opposite was true when the goal of eating more fruits and vegetables was presented as a health prevention-focused issue, and no main effects of regulatory focus or outcome framing were identified.

Experiment two was notable, where participants were requested to read either a promotion or a prevention-framed health message urging them to eat more fruits and vegetables; a goal was explicitly set. In line with regulatory focus theory and our model, we argue that social influence works best when targeting either a promotion (approach) or a prevention (avoidance) feedback loop in the target. Researchers could also directly address action selection by giving participants specific action planning options in addition to the goal state (imagination of benefits). Accordingly, a fit between influencing action selection ( *e.g.,* identifying a good time) and influencing the goal ( *i.e.,* outcome benefits) may provide the best results in influencing action selection. Future research might manipulate feedback prediction by adding comparative information about the degree and likelihood of success toward dietary change.

## Advertisement

Zhao and Pechmann (2007) examined the impact of regulatory focus and different framing strategies on the persuasiveness of antismoking advertisements for high school students. This study illustrates how behavior can be influenced during action selection, but also the goal state and the feedback prediction according to our framework. The authors created four antismoking advertisements that differed in their regulatory focus (promotion versus prevention) and outcome framing (favorable versus unfavorable). In the first experiment, the authors used a three-factor between-design categorization, classifying participants based on their dominant regulatory focus (assessed via a validated scale), the advertisement’s regulatory focus, and the outcome framing. A control group with a non-smoking-related advertisement was included. The criterion was the intention not to smoke cigarettes. The study found that anti-smoking advertisements were most effective when the viewers’ regulatory focus, the advertisement’s regulatory focus, and the message framing worked together. Specifically, promotion-focused individuals responded best to promotion-focused advertisements with a positive outcome framing, while prevention-focused individuals responded best to prevention-focused advertisements with a negative outcome framing. A second experiment with a similar design, but with an active manipulation of participants’ regulatory focus, replicated the results of the first experiment.

The participants in this study were randomly assigned to groups and watched an episode of a TV show where the advertisements were shown between several other filler advertisements. The goal to be implemented or strengthened in targets was non-smoking, which might be pursued by addressing health issues. However, advertisements also specifically added social goals to the context of smoking, particularly social acknowledgment and attachment. Thus, the goal level was directly addressed with these advertisements; the social environment provided positive and negative feedback toward the depicted smoker.

Per our framework, the target of the presented experiments in Zhao and Pechmann (2007) was to manipulate behavior ( *i.e.,* the post-experimental questionnaire assessing the intention to smoke) *via* the presentation of these advertisements. The inclusion of the advice, “Don’t smoke!” in the advertisement had a direct influence on action selection but did not seem to change behavior compared to the control group. Feedback prediction was influenced by showing participants the potential social consequences of smoking and non-smoking through the behavior present in the advertisement’s social context. The study clearly showed that a match among individual regulatory focus, outcome type, and the regulatory or motivational state leads to the greatest changes in behavior. In the feedback loop framework, we argue that manipulation works on a specific feedback loop in a target. Ideally, all manipulation aspects should converge on the same feedback loop—either a promotion/approach loop or a prevention/avoidance loop.

Several options can be employed to extend the experimental design and add manipulation possibilities using our framework. Although Zhao and Pechmann (2007) included a control condition for the task, all participants were classified by a regulatory focus or induction. An additional, unbiased control group without regulatory focus induction should be included in future research. Furthermore, the intention to smoke may vary in contexts outside the school classroom. Therefore, future studies could examine the influence of situational factors, such as watching a TV show in the classroom and on a smartphone in the corner of the schoolyard. Situational influences on intentions might then be examined by measuring baseline intentions to smoke and previous smoking behavior; these state variables might interact with the implementation of the social goals using the advertisements. In smokers or people with stronger smoking intentions, social goal implementation likely leads to a stronger is-ought discrepancy, which may increase adjustments or reactance; for example, if the discrepancy is too large for an individual to deal with. Future research should assess the level of is-ought behavior discrepancy.

## Nudging

According to Thaler and Sunstein (2008), nudging refers to interventions implemented by persons with responsibilities over a group to help those individuals make responsible decisions. We used a study investigating the effects of nudging on when and why users opt for online ID verification in the context of digital platforms to explore the application of our model in an example ( Schneider *et al.*, 2017). This study is a good case of targeting action selection rather than goal structures. The researchers combined assurance statements as claims (which were framed as promotion and prevention focus) on a fictional car-sharing platform. The supporting data covered the convenience, security, and privacy aspects of online verification (present versus absent). The dependent variable was the choice for online verification ( *i.e.,* a webcam session with a verification agent) compared with offline verification ( *e.g.,* physically visiting a post office). A control group without claims and data was included in addition to the four experimental groups (two claims, each with present or absent data). The results showed that participants in the promotion focus group were more likely to choose online verification when no data was available than those in the prevention focus group. However, this effect was reversed in the presence of data, as participants chose online verification more often in the prevention focus group than in the promotion focus group.

Concerning the goal state of achieving verification, two paths of action were available. Regarding action selection, participants faced a binary choice: online versus offline verification. They were asked to put themselves in the position of a prospective car-sharing user, and the goal of completing the registration process was implemented. In this case, the goal value was not manipulated, but the means to achieve it was ( *i.e.*, online versus offline verification). Depending on the car-sharing platform’s relevance for participants, their personal goals might have amplified the goal state. For example, participants who used car-sharing platforms more often were motivated to participate. A general bias towards a digital context might have implicitly influenced the situation because the study was conducted online, and participants were asked to take the perspective of a car-sharing user. The manipulation of action selection was targeted by increasing the credibility of online verification by including security-related data on the platform and the regulatory focus framing of the claims.

By conducting the experiment in a laboratory, researchers could manipulate situational factors—such as conducting offline verification in a controlled manner and in a separate room—which reduced the influence of the distance between participants’ homes and a post office. To manipulate action, a queue condition could be implemented in the offline verification process, highlighting the time-related advantages of the online process. To influence feedback prediction, the researchers could use a sequential design to present regulatory focus framing and supportive security data, evaluate the choice option multiple times, and provide participants with more information about the likelihood of success and the difficulties in attaining the goal.

We further selected a series of studies on dietary decisions to apply our model ( Prinsen *et al.*, 2013). The rationale of Prinsen *et al.* (2013) was that individuals conform to the eating behavior of others and, therefore, should be affected by the corresponding environmental cues that signal what others have done. The authors conducted field and laboratory experiments using a between-group design with a bowl of hand-wrapped chocolates. The amount of chocolate taken was the outcome variable. The researchers placed a second bowl next to the bowl of hand-wrapped chocolates; this second bowl had twenty used wrappers for one group of participants and was empty for the second group. Participants were more likely to take chocolates in the presence of an environmental cue ( *i.e.,* empty wrappers indicating that previous participants also took chocolate). In another experiment, the authors manipulated the healthiness of the snack (healthy snack compared with unhealthy snack) and the goal prime (a magazine on healthy eating versus a magazine on hedonic eating) to measure snack choice. The results indicated that participants conformed to environmental cues about the food type others had eaten in addition to the eating behavior of others in general. The participants in all experiments were given a false story that they were waiting for the experiment to start; the authors evaluated the experiments during this waiting period.

In the first two experiments, no food-related goal state was implemented or directly manipulated for participants. However, since participants had to answer several questions ( *e.g.,* the time difference to their last meal), we cannot completely rule out that at least some of the participants focused on the food. Participants in the laboratory settings were told that the study assessed their reaction time (Experiment two) or their completion of a cognitive task (Experiment three). The empty wrappers may have worked to manipulate the situation in several ways: they may disinhibit eating behavior because other participants had already eaten the chocolate, which could activate social motivation to act similarly (activating a social goal state). Second, they may have directly influenced action selection by promoting simple behavioral imitation.

To manipulate the goal state, a possible experimental condition would be to label the fake “target” tasks as food-related or not. In the published study, the authors focused on manipulating the situation with the filled and empty bowls, which indicated past participants’ behavior and demonstrated that such behavior impacted decision-making. Moreover, the magazines on healthy versus hedonic eating might be considered manipulation tactics influencing the goal state or action selection. These magazines should only affect behavior if the decision to take food has already been made. In this task, manipulating the action could pose ethical issues involving forcing someone to eat. However, this may be accomplished by experiments relating to tasting and judging food, which makes people eat. Implementing a prediction error in the feedback loop of the participants would suffice to manipulate feedback prediction. For example, if the instruction had been that “eating is voluntary, but healthy versus unhealthy food (or *vice versa*) that does not get eaten will be thrown away,” they might have experienced a conflict between the behavior of previous participants, the goal prime induced by the magazine they read, and the type of food that would be discarded. In addition, the subjective situation may be manipulated by letting participants enter the experiment in a hungry or a full state.

To ensure the optimal nudging practice, we suggest that it may be critical to reducing the discrepancy between the optimal outcome (choosing more healthy food) and the initial goal state. To disentangle the indirect influences on goal and direct influences on action, one option could be to use videos that depict or do not depict eating behavior. Additionally, the videos could include a verbal message promoting or preventing a certain kind of eating behavior, such as unhealthy food. While the direct influence on action may be the imitation of the seen/condemned behavior, the impact on the goal state should depend on the verbal content of the material promoting health. Additionally, all levels of manipulation might address either a promotion or a prevention feedback loop.

## Boundary conditions

We deal with apparent boundary conditions to complete the theoretical discourse of the social influence on a target’s feedback loop system. Our proposed model, and probably influence in general, depends on the reactance of the *target.* Some people may be more susceptible to influence strategies than others, and these differences may hinder or even exclude the success of social influence. Furthermore, the ability of the *target* to mentalize the *influencer*’s intentions may interfere with the *influencer’s* successful exertion of influence. Therefore, highly reactant individuals and people good at mentalizing their social interaction partners might not be good *targets* for applying this framework. However, it may be interesting to examine whether the discrepancy between the reference or goal state and the current state or situation can predict reactance. Potentially, a discrepancy that seems too large for the individual may promote reactance. Alternatively, there may be a threshold at which the magnitude of the discrepancy between the goal state and the current state renders the pursuit of the goal obsolete, thus resulting in a decline in reactance.

## Conclusions

We proposed a social influence model that targets different parts of an interaction partner’s internal feedback loop. To illustrate the need for this model, we explained the influence on the goal state, action selection, action, and feedback prediction in detail. Lastly, we analyzed classical experiments on social influence, conformity, and advertisements and applied our model. As many examples have shown, there are different ways to enter another person’s feedback loop by exerting external social influence. Depending on the *influencer’s* intentions, one specific influence tactic might affect different stages or even more than one stage of the cybernetic loop system at the same time.

With our proposed model, we aimed to present a new perspective on classic experiments and paradigms that have investigated different kinds of social influence and to classify them according to our model. Future studies should selectively examine the single stages of social influence and combine this model with others, such as reinforcement learning. In addition, future research should carefully consider whether there are interindividual differences in the influenced person, such as gender or personality, that might promote or hinder the effectiveness of different influence strategies according to our model. In our view, understanding how to influence only one particular aspect while excluding the influence of another aspect may be crucial for a more detailed perspective on social influence.

## Data Availability

No data are associated with this article.

## Footnotes

^1^

Schultz *et al.* (2008) could not replicate differences between the two types of normative messages. Hence, further research is needed to investigate conditions under which different types of messages increase pro-environmental behavior in this context.

## References

[ref1] Aaker JL Lee AY : “I” seek pleasures and “we” avoid pains: The role of self-regulatory goals in information processing and persuasion. *J. Consum. Res.* 2001;28(1):33–49. 10.1086/321946

[ref2] Aiello A Tesi A Pratto F : Social dominance and interpersonal power: Asymmetrical relationships within hierarchy-enhancing and hierarchy-attenuating work environments. *J. Appl. Soc. Psychol.* 2018;48(1):35–45. 10.1111/jasp.12488

[ref97] Albert D Chein J Steinberg L : The teenage brain: Peer influences on adolescent decision making. *Curr. Dir. Psychol. Sci.* 2013;22(2):114–120. 10.1177/0963721412471347 25544805 PMC4276317

[ref3] Asch SE : Studies in the principles of judgments and attitudes: II. Determination of judgments by group and by ego standards. *J. Soc. Psychol.* 1940;12(2):433–465. 10.1080/00224545.1940.9921487

[ref4] Bednark JG Franz EA : Agency attribution: Event-related potentials and outcome monitoring. *Exp. Brain Res.* 2014;232(4):1117–1126. 10.1007/s00221-014-3821-4 24504195

[ref5] Behne N Scheich H Brechmann A : The left dorsal striatum is involved in the processing of neutral feedback. *NeuroReport.* 2008;19(15):1497–1500. 10.1097/WNR.0b013e32830fe98c 18797305

[ref6] Brown VJ Bowman EM : Discriminative cues indicating reward magnitude continue to determine reaction time of rats following lesions of the nucleus accumbens. *Eur. J. Neurosci.* 1995;7(12):2479–2485. 10.1111/j.1460-9568.1995.tb01046.x 8845953

[ref7] Bruins J : Social power and influence tactics: A theoretical introduction. *J. Soc. Issues.* 1999;55(1):7–14. 10.1111/0022-4537.00101

[ref8] Buss DM : Manipulation in close relationships: Five personality factors in interactional context. *J. Pers.* 1992;60(2):477–499. 10.1111/j.1467-6494.1992.tb00981.x 1635051

[ref9] Carver CS : Self-regulation of action and affect. Vohs KD Baumeister RF , editors. *Handbook of self-regulation: Research, theory, and applications.* The Guilford Press;2004; pp.13–39.

[ref10] Carver CS Scheier MF : *Attention and self-regulation: A control-theory approach to human behavior.* New York: Springer-Verlag;1981. 10.1007/978-1-4612-5887-2

[ref11] Carver CS Scheier MF : Scaling back goals and recalibration of the affect system are processes in normal adaptive self-regulation: Understanding “response shift” phenomena. *Soc. Sci. Med.* 2000a;50(12):1715–1722. 10.1016/S0277-9536(99)00412-8 10798327

[ref12] Carver CS Scheier MF : On the structure of behavioral self-regulation. Boekaerts M Pintrich PR Zeidner M , editors. *Handbook of self-regulation.* Academic Press;2000b; pp.41–84. 10.1016/b978-012109890-2/50032-9

[ref98] Caspar EA Beyer F Cleeremans A : The obedient mind and the volitional brain: A neural basis for preserved sense of agency and sense of responsibility under coercion. *PLoS One.* 2021;16(10):e0258884. 10.1371/journal.pone.0258884 34710149 PMC8553174

[ref13] Cesario J Higgins ET : Making message recipients “feel right”: How nonverbal cues can increase persuasion. *Psychol. Sci.* 2008;19(5):415–420. 10.1111/j.1467-9280.2008.02102.x 18466399

[ref14] Chambon V Haggard P : Sense of control depends on fluency of action selection, not motor performance. *Cognition.* 2012;125(3):441–451. 10.1016/j.cognition.2012.07.011 22902284

[ref15] Chang CC Chou YJ : Goal orientation and comparative valence in persuasion. *J. Advert.* 2008;37(1):73–87. 10.2753/JOA0091-3367370106

[ref16] Cialdini RB Goldstein NJ : Social influence: Compliance and conformity. *Annu. Rev. Psychol.* 2004;55:591–621. 10.1146/annurev.psych.55.090902.142015 14744228

[ref17] Cialdini RB Reno RR Kallgren CA : A focus theory of normative conduct: Recycling the concept of norms to reduce littering in public places. *J. Pers. Soc. Psychol.* 1990;58(6):1015–1026. 10.1037/0022-3514.58.6.1015

[ref18] Cullum J Armeli S Tennen H : Drinking norm-behavior association over time using retrospective and daily measures. *J. Stud. Alcohol Drugs.* 2010;71(5):769–777. 10.15288/jsad.2010.71.769 20731984 PMC2930509

[ref19] Deutsch M Gerard HB : A study of normative and informational social influences upon individual judgment. *J. Abnorm. Soc. Psychol.* 1955;51(3):629–636. 10.1037/h0046408 13286010

[ref20] Diel K Grelle S Hofmann W : A motivational framework of social comparison. *J. Pers. Soc. Psychol.* 2021;120:1415–1430. 10.1037/pspa0000204 33507785

[ref21] Do KT Prinstein MJ Telzer EH : Neurobiological susceptibility to peer influence in adolescence. Cohen Kadosh K , editors. *The Oxford Handbook of Developmental Cognitive Neuroscience.* Oxford University Press;2022. 10.1093/oxfordhb/9780198827474.013.27

[ref22] Druckman JN : Using credible advice to overcome framing effects. *J. Law Econ. Org.* 2001;17(1):62–82. 10.1093/jleo/17.1.62

[ref23] Elsner B Hommel B : Effect anticipation and action control. *J. Exp. Psychol. Hum. Percept. Perform.* 2001;27(1):229–240. 10.1037/0096-1523.27.1.229 11248937

[ref24] Falk E Scholz C : Persuasion, influence, and value: Perspectives from communication and social neuroscience. *Annu. Rev. Psychol.* 2018;69:329–356. 10.1146/annurev-psych-122216-011821 28961060 PMC12175252

[ref25] Fischer AH Manstead ASR Zaalberg R : Social influences on the emotion process. *Eur. Rev. Soc. Psychol.* 2003;14(1):171–201. 10.1080/10463280340000054

[ref26] Fiske ST : Controlling other people: The impact of power on stereotyping. *Am. Psychol.* 1993;48(6):621–628. 10.1037/0003-066X.48.6.621 8328729

[ref27] Freedman JL Fraser SC : Compliance without pressure: The foot-in-the-door technique. *J. Pers. Soc. Psychol.* 1966;4(2):195–202. 10.1037/h0023552 5969145

[ref28] French JRP Raven B : The bases of social power. Cartwright D , editor. *Studies in social power.* University of Michigan;1959; pp.150–167.

[ref29] Frick TW Li T Pavlou P : Social influence and visual attention in the personalization privacy paradox for social advertising: An eye tracking study. *WISP 2018 Proceedings.* 2018;8.

[ref100] Gass RH Seiter JS : *Persuasion: Social influence and compliance gaining.* Routledge;2022. 10.4324/9781003081388

[ref30] Gächter S Orzen H Renner E : Are experimental economists prone to framing effects? A natural field experiment. *J. Econ. Behav. Organ.* 2009;70(3):443–446. 10.1016/j.jebo.2007.11.003

[ref31] Goldstein NJ Cialdini RB Griskevicius V : A room with a viewpoint: Using social norms to motivate environmental conservation in hotels. *J. Consum. Res.* 2008;35(3):472–482. 10.1086/586910

[ref32] Haddock G Maio GR Arnold K : Should persuasion be affective or cognitive? The moderating effects of need for affect and need for cognition. *Personal. Soc. Psychol. Bull.* 2008;34(6):769–778. 10.1177/0146167208314871 18344496

[ref33] Harkins SG Williams KD Burger JM : *The Oxford handbook of social influence.* Oxford University Press;2017. 10.1093/oxfordhb/9780199859870.001.0001

[ref101] Haslam SA Reicher SD Birney ME : Nothing by mere authority: Evidence that in an experimental analogue of the Milgram paradigm participants are motivated not by orders but by appeals to science. *J. Soc. Issues.* 2014;70(3):473–488. 10.1111/josi.12072

[ref34] Higgins CA Judge TA Ferris GR : Influence tactics and work outcomes: A meta-analysis. *J. Organ. Behav.* 2003;24(1):89–106. 10.1002/job.181

[ref35] Higgins ET : Beyond pleasure and pain. *Am. Psychol.* 1997;52(12):1280–1300. 10.1037/0003-066x.52.12.1280 9414606

[ref36] Higgins ET : Promotion and Prevention: Regulatory Focus as A Motivational Principle. *Adv. Exp. Soc. Psychol.* 1998;30:1–46. 10.1016/S0065-2601(08)60381-0

[ref37] Higgins ET : Promotion and prevention as a motivational duality: Implications for evaluative processes. Chaiken S Trope Y , editors. *Dual-process theories in social psychology.* The Guilford Press;1999; pp.503–525. Reference Source

[ref102] Hollander MM Turowetz J : Normalizing trust: Participants’ immediately post-hoc explanations of behaviour in Milgram’s ‘obedience’experiments. *Br. J. Soc. Psychol.* 2017;56(4):655–674. 10.1111/bjso.12206 28653413

[ref38] Holler M Hoelzl E Kirchler E : Framing of information on the use of public finances, regulatory fit of recipients and tax compliance. *J. Econ. Psychol.* 2008;29(4):597–611. 10.1016/j.joep.2008.01.001 20495689 PMC2874666

[ref39] Hommel B Elsner B : Acquisition, representation, and control of action. Morsella E Bargh JA Gollwitzer PM , editors. *Social cognition and social neuroscience. Oxford handbook of human action.* Oxford University Press;2009; pp.371–398.

[ref40] Jäger A Loschelder DD Friese M : How self-regulation helps to master negotiation challenges: An overview, integration, and outlook. *Eur. Rev. Soc. Psychol.* 2015;26(1):203–246. 10.1080/10463283.2015.1112640

[ref103] Lammers J Stoker JI Rink F : To have control over or to be free from others? The desire for power reflects a need for autonomy. *Pers. Soc. Psychol. Bull.* 2016;42(4):498–512. 10.1177/0146167216634064 26984014

[ref41] Lavine H Snyder M : Cognitive processing and the functional matching effect in persuasion: The mediating role of subjective perceptions of message quality. *J. Exp. Soc. Psychol.* 1996;32(6):580–604. 10.1006/jesp.1996.0026 8979935

[ref42] Lee AY Aaker JL : Bringing the frame into focus: The influence of regulatory fit on processing fluency and persuasion. *J. Pers. Soc. Psychol.* 2004;86(2):205–218. 10.1037/0022-3514.86.2.205 14769079

[ref104] Legros S Cislaghi B : Mapping the social-norms literature: An overview of reviews. *Perspect. Psychol. Sci.* 2020;15(1):62–80. 10.1177/1745691619866455 31697614 PMC6970459

[ref43] Leonard A Scholte T Shepherd K : Cybernetics Approaches and Models. Metcalf GS Kijima K Deguchi H , editors. *Handbook of Systems Sciences.* Springer;2021; pp.67–86. 10.1007/978-981-15-0720-5_66

[ref44] Leotti LA Delgado MR : The inherent reward of choice. *Psychol. Sci.* 2011;22(10):1310–1318. 10.1177/0956797611417005 21931157 PMC3391581

[ref45] Levi D Askay DA : *Group dynamics for teams.* SAGE Publications;2015.

[ref46] Lewin K : Analysis of the concepts whole, differentiation, and unity. *University of Iowa Studies in Child Welfare.* 1941;18(1):226–261.

[ref47] Mansell W : Changing Behavior Using Control Theory. Hamilton K Cameron LD Hagger MS , editors. *The Handbook of Behavior Change.* Cambridge University Press;2020; pp.120–135. 10.1017/9781108677318.009

[ref48] Marsh KL Hart-O’Rourke DM Julka DL : The Persuasive Effects of Verbal and Nonverbal Information in a Context of Value Relevance. *Personal. Soc. Psychol. Bull.* 1997;23(6):563–579. 10.1177/0146167297236001

[ref49] McClelland KA Fararo TJ : *Purpose, meaning, and action: Control systems theories in sociology.* Palgrave-MacMillan;2006.

[ref50] McDonald RI Crandall CS : Social norms and social influence. *Curr. Opin. Behav. Sci.* 2015;3:147–151. 10.1016/j.cobeha.2015.04.006

[ref51] Melnyk V Herpen E van Fischer ARH : Regulatory fit effects for injunctive versus descriptive social norms: Evidence from the promotion of sustainable products. *Mark. Lett.* 2013;24(2):191–203. 10.1007/s11002-013-9234-5

[ref52] Milgram S : Behavioral study of obedience. *J. Abnorm. Soc. Psychol.* 1963;67(4):371–378. 10.1037/h0040525 14049516

[ref53] Min KS Martin D Jung JM : Designing advertising campaigns for destinations with mixed images: Using visitor campaign goal messages to motivate visitors. *J. Bus. Res.* 2013;66(6):759–764. 10.1016/j.jbusres.2011.09.015

[ref54] Moussaïd M Kämmer JE Analytis PP : Social Influence and the Collective Dynamics of Opinion Formation. *PLoS One.* 2013;8(11):e78433. 10.1371/journal.pone.0078433 24223805 PMC3818331

[ref55] Nelson TE Kinder DR : Issue frames and group-centrism in American public opinion. *J. Polit.* 1996;58(4):1055–1078. 10.2307/2960149

[ref56] Nelson TE Oxley ZM Clawson RA : Toward a psychology of framing effects. *Polit. Behav.* 1997;19(3):221–246. 10.1023/A:1024834831093

[ref57] Pentina I Taylor DG : Regulatory Focus and Daily-Deal Message Framing: Are We Saving or Gaining With Groupon? *J. Interact. Advert.* 2013;13(2):67–75. 10.1080/15252019.2013.791792

[ref58] Petty RE Brinol P Loersch C : The need for cognition. Leary R Hoyle RH , editors. *Handbook of individual differences in social behavior.* The Guilford Press;2009; pp.318–329. Reference Source

[ref59] Petty RE Cacioppo JT : *Communication and persuasion: Central and peripheral routes to attitude change.* Springer;1986. 10.1007/978-1-4612-4964-1

[ref60] Petty RE Wegener DT : Matching Versus Mismatching Attitude Functions: Implications for Scrutiny of Persuasive Messages. *Personal. Soc. Psychol. Bull.* 1998;24(3):227–240. 10.1177/0146167298243001

[ref61] Poels K Dewitte S : Hope and self-regulatory goals applied to an advertising context. Promoting prevention stimulates goal-directed behavior. *J. Bus. Res.* 2008;61(10):1030–1040. 10.1016/j.jbusres.2007.09.019

[ref62] Price V Tewksbury D : News values and public opinion: A theoretical account of media priming and framing. Barett GA Boster FJ , editors. *Progress in the communication sciences.* Vol. 13. Ablex;1997; pp.173–212.

[ref63] Prinsen S Ridder DTD de Vet E de : Eating by example. Effects of environmental cues on dietary decisions. *Appetite.* 2013;70:1–5. 10.1016/j.appet.2013.05.023 23791633

[ref64] Raven BH : *Social Influence and Power.* California Univ;1964.

[ref65] Raven BH : A power/interaction model of interpersonal influence: French and Raven thirty years later. *J. Soc. Behav. Pers.* 1992;7(2):217–244.

[ref66] Redgrave P Gurney K : The short-latency dopamine signal: A role in discovering novel actions? *Nat. Rev. Neurosci.* 2006;7(12):967–975. 10.1038/nrn2022 17115078

[ref67] Redgrave P Gurney K Reynolds J : What is reinforced by phasic dopamine signals? *Brain Res. Rev.* 2008;58(2):322–339. 10.1016/j.brainresrev.2007.10.007 18055018

[ref68] Redgrave P Prescott TJ Gurney K : The basal ganglia: A vertebrate solution to the selection problem? *Neuroscience.* 1999;89(4):1009–1023. 10.1016/S0306-4522(98)00319-4 10362291

[ref105] Ren Q Gentsch A Kaiser J : Ready to go: higher sense of agency enhances action readiness and reduces response inhibition. *Cognition.* 2023;237:105456. 10.1016/j.cognition.2023.105456 37037164

[ref69] Robinson DT : Control theories in sociology. *Annu. Rev. Sociol.* 2007;33:157–174. 10.1146/annurev.soc.32.061604.123110

[ref70] Sadiq M Adil M Paul J : Does social influence turn pessimistic consumers green? *Bus. Strateg. Environ.* 2021;30(7):2937–2950. 10.1002/bse.2780

[ref71] Samejima K Ueda Y Doya K : Representation of action-specific reward values in the striatum. *Science.* 2005;310(5752):1337–1340. 10.1126/science.1115270 16311337

[ref72] Schneider D Lins S Grupp T : Nudging users into online verification: The case of carsharing platforms. *ICIS 2017: Transforming Society with Digital Innovation.* 2017.

[ref73] Schultz WP Khazian AM Zaleski AC : Using normative social influence to promote conservation among hotel guests. *Soc. Influ.* 2008;3(1):4–23. 10.1080/15534510701755614

[ref74] Sherif M : *The psychology of social norms.* Harper & Brothers;1936.

[ref75] Slater M Antley A Davison A : A virtual reprise of the Stanley Milgram obedience experiments. *PLoS One.* 2006;1(1):e39. 10.1371/journal.pone.0000039 17183667 PMC1762398

[ref106] Sosa M Giocomo LM : Navigating for reward. *Nat. Rev. Neurosci.* 2021;22(8):472–487. 10.1038/s41583-021-00479-z 34230644 PMC9575993

[ref107] Spears R : Social influence and group identity. *Annu. Rev. Psychol.* 2021;72:367–390. 10.1146/annurev-psych-070620-111818 32931718

[ref76] Spiegel S Grant-Pillow H Tory Higgins E : How regulatory fit enhances motivational strength during goal pursuit. *Eur. J. Soc. Psychol.* 2004;34(1):39–54. 10.1002/ejsp.180

[ref77] Stayton DJ Hogan R Ainsworth MD : Infant obedience and maternal behavior: The origins of socialization reconsidered. *Child Dev.* 1971;42(4):1057–1069. 10.2307/1127792 5157102

[ref78] Sunstein CR : Nudging: A Very Short Guide. *J. Consum. Policy.* 2014;37(4):583–588. 10.1007/s10603-014-9273-1

[ref79] Sutton RS Barto AG : Reinforcement Learning: An Introduction. *IEEE Transactions on Neural Networks.* Vol.9. MIT Press;1998. 10.1109/tnn.1998.712192

[ref80] Thaler RH Sunstein CR : *Nudge: Improving decisions about health, wealth, and happiness.* Yale University Press;2008.

[ref81] Thibaut JW Kelley HH : *The social psychology of groups.* John Wiley & Sons, Inc.;1959.

[ref82] Tricomi EM Delgado MR Fiez JA : Modulation of caudate activity by action contingency. *Neuron.* 2004;41(2):281–292. 10.1016/S0896-6273(03)00848-1 14741108

[ref83] Turner JC : *Social influence.* Thomson Brooks/Cole Publishing Co;1991;xvi, 206.

[ref84] Tversky A Kahneman D : The framing of decisions and the psychology of choice. *Science.* 1981;211(4481):453–458. 10.1126/science.7455683 7455683

[ref85] vanDellen MR Hoyle RH : Regulatory accessibility and social influences on state self-control. *Personal. Soc. Psychol. Bull.* 2010;36(2):251–263. 10.1177/0146167209356302 20008967 PMC3664398

[ref108] Van Dijke M Poppe M : Striving for personal power as a basis for social power dynamics. *Eur. J. Soc. Psychol.* 2006;36(4):537–556. 10.1002/ejsp.351

[ref86] Vansteenkiste M Simons J Lens W : Motivating learning, performance, and persistence: The synergistic effects of intrinsic goal contents and autonomy-supportive contexts. *J. Pers. Soc. Psychol.* 2004;87(2):246–260. 10.1037/0022-3514.87.2.246 15301630

[ref87] Vescio TK Butz DA Snyder M : Power in stereotypically masculine domains: A Social Influence Strategy × Stereotype Match Model. *J. Pers. Soc. Psychol.* 2003;85(6):1062–1078. 10.1037/0022-3514.85.6.1062 14674814

[ref88] Bertalanffy L von : An outline of general system theory. *Br. J. Philos. Sci.* 1950;1(2):134–165. 10.1093/bjps/I.2.134

[ref89] Hippel W von Trivers R : Reflections on self-deception. *Behav. Brain Sci.* 2011;34(1):41–56. 10.1017/S0140525X10003018 21288379

[ref90] Wheeler L : Motivation as a determinant of upward comparison. *J. Exp. Soc. Psychol.* 1966;1(Suppl. 1):27–31. 10.1016/0022-1031(66)90062-X

[ref91] Wiener N : Cybernetics: Control and communication in the animal and the machine. *Econ. J.* 1949;59(236):573–575. 10.2307/2226579

[ref92] Willer R Kuwabara K Macy MW : The false enforcement of unpopular norms. *Am. J. Sociol.* 2009;115(2):451–490. 10.1086/599250 20614762

[ref93] Wills TA : Downward comparison principles in social psychology. *Psychol. Bull.* 1981;90(2):245–271. 10.1037/0033-2909.90.2.245

[ref94] Wolpert DM Doya K Kawato M : A unifying computational framework for motor control and social interaction. *Philos. Trans. R. Soc. B: Biol. Sci.* 2003;358(1431):593–602. 10.1098/rstb.2002.1238 PMC1693134 12689384

[ref109] Wolske KS Gillingham KT Schultz PW : Peer influence on household energy behaviours. *Nat. Energy.* 2020;5(3):202–212. 10.1038/s41560-019-0541-9

[ref95] Wood W : Attitude change: Persuasion and social influence. *Annu. Rev. Psychol.* 2000;51(1):539–570. 10.1146/annurev.psych.51.1.539 10751980

[ref96] Zhao G Pechmann C : The impact of regulatory focus on adolescents’ response to antismoking advertising campaigns. *J. Mark. Res.* 2007;44(4):671–687. 10.1509/jmkr.44.4.671

