# Supplementary material for: Social influence and external feedback control in humans
Source: F1000Res. 2024 Feb 16;12:438. Originally published 2023 Apr 24. [Version 3] doi: 10.12688/f1000research.133295.3 (PMC10905169; doi:10.12688/f1000research.133295.3)
Supplement: Peer review history

# Reviewer report: Reviewer response for version 2

**DOI:** 10.5256/f1000research.160513.r235517

**Published:** 2024-02-08

**Authors:** Robin Willardt

**Affiliations:** 1 University of New South Wales, Sydney, New South Wales, Australia

On page 6, there is a syntax error:

 "We do not aim at conceptualizing persuasion is a sub-facet of influence"

 On page 8/9, the authors write "Persuasive messages targeting important attitude functions are processed carefully and are less influential". Could it be that the authors mean "...more influential"?

 On page 11 the authors write "According to our model, we argued that this was due to the inclusion and presence of a social goal". I suggest to use present tense ("we argue") for consistency.

 Apart from the minor points above, I have no further comments to make.

Is the topic of the opinion article discussed accurately in the context of the current literature?

Yes

Are arguments sufficiently supported by evidence from the published literature?

Yes

Are all factual statements correct and adequately supported by citations?

Partly

Are the conclusions drawn balanced and justified on the basis of the presented arguments?

Yes

Reviewer Expertise:

Social psychology, motivation, affect, approach-/avoidance-motivation, anger, powerlessness, threats

I confirm that I have read this submission and believe that I have an appropriate level of expertise to confirm that it is of an acceptable scientific standard.

# Reviewer report: Reviewer response for version 2

**DOI:** 10.5256/f1000research.160513.r235518

**Published:** 2024-01-23

**Authors:** Yanyan Qi

**Affiliations:** 1 School of Education, Zhengzhou University, Zhengzhou, Henan, China

I thank the authors' work, I have no further comments.

Is the topic of the opinion article discussed accurately in the context of the current literature?

Yes

Are arguments sufficiently supported by evidence from the published literature?

Yes

Are all factual statements correct and adequately supported by citations?

Yes

Are the conclusions drawn balanced and justified on the basis of the presented arguments?

Partly

Reviewer Expertise:

Social psychology, social behavior, and social emotions

I confirm that I have read this submission and believe that I have an appropriate level of expertise to confirm that it is of an acceptable scientific standard.

# Reviewer report: Reviewer response for version 1

**DOI:** 10.5256/f1000research.146274.r218073

**Published:** 2023-11-21

**Authors:** Robin Willardt

**Affiliations:** 1 University of New South Wales, Sydney, New South Wales, Australia

Thank you for the opportunity to review the opinion article “Social influence and external feedback control in humans”.

 In the article, the authors use a feedback loop model to categorize and describe the different ways in which an influencing person can exert social influence on a targeted person. Most of my comments are concerned with the clarity of the article. In the following, I will outline my feedback in detail following the structure of the paper. Afterwards, I emphasize two points in particular that should be addressed: The justification for introducing the suggested feedback loop model and the nomenclature used to derive at the model.

 The sentence “Different approaches have shown that social influence is related to emotional processes, visual attention, and self regulation” (page 3) feels like the justification for connecting a feedback loop model with social influence. If that is indeed the case, this should be stated more explicitly.

 The term feedback control on page 3 (“In social interactions, actions elicit responses from others, leading to the possibility of feedback control…”) should be explained in greater detail.

 On page 4 under the heading “Social influence”, a sentence is started with the word ‘nonetheless’, even though the preceding sentence does not entail a contradiction to the subsequent one.

 It might be interesting for the discussion of social influence on page 4 that there is some power research indicating that power actually consists of two subcomponents, namely personal control and influence over others (e.g., Lammers et al, 2016; Van Dijke & Poppe, 2006).

 When describing the individual components of the feedback loop model, it would add clarity to specifically state whose goals are meant with the term “goal state”. Those of the target of those of the influencer? The same goes for the feedback prediction component.

 Is the comparator/reference component the plus/minus sign in the graphical depiction of the model? Please clarify.

 On page 4 the authors introduce the struggle for a TV to illustrate the model. The goal in this case is described as “the division of a shared television”. But this seems to represent a communal goal rather than the individual goals of the two roommates. Maybe it would be better to avoid the term “goal” in this situation and only use it to refer to the target’s goals in the examples that follows.

 In the subsequent sentence, the goal state is described as the target buying the television. The use of goal and goal state and the lack of a clear attribution of these terms to either one of the two roommates is very confusing.

 See also the sentence “In this situation, the influencer might persuade the target that the goal is not worthwhile” where it is unclear what goal the authors refer to.

 On page 5, the heading reads “Social influence towards the goal state”. This is somewhat irritating since social influence towards the goal state was already touched upon in the example of the two roommates (the same applies to the subsequent headings).

 Here, the authors should also explain why a differentiation between persuasion and manipulation is necessary.

 The sentence “Influence through persuasion can affect the goal state even before the target takes any action. Persuasion is the most appropriate term for what we consider influence toward the goal state” is hard to comprehend: At this point it is yet unclear what the difference between influence and persuasion is. Is persuasion a sub-facet of influence, namely influence towards the goal state? Please explain.

 The terms ‘message elaboration’ and ‘peripheral route’ should also be outlined in greater detail

 The authors refer to Sherif’s experiment (p. 7). Outlining the mentioned goal the manipulator is thought to influence (I assume it is the social goal to give group-conform responses) would help to better imbed this research.

 Under the heading “Social influence on the subjective situation” on page 7, the authors refer to social comparison. The connection of this concept to the example given and to social influence on the situation remains somewhat unclear.

 On page 8, the authors write “In the context of the shared TV example, imagine that the influencer wants to donate the TV to charity, but the target wants to keep it.” In that case the goal state of the target would be to keep the TV. I suggest to emphasize the connection between the used examples (such as this one) and the components of the proposed model more (in this case: target’s goal à keep TV).

 Also on page 8, the authors write “If the target perceives the influencer as highly reputable and trustworthy, they may be more likely to obey or comply with the influencer’s advice. This is true, even if the advice does not necessarily align with the target’s intuitive approach to achieving their goals.” This empirical claim should be backed up by a reference.

 Under the heading “Social influence on behavior while acting”, the authors write “The social influence induced by social norms can alter the internal feedback loop regarding action selection.” This implies that the authors refer to the subcomponent action selection rather than the subcomponent action here (see also the subsequent sentence). It would help the clarity of the paper to precisely state which subcomponents the authors target.

 Under the heading “Social influence on feedback prediction”, the authors outline the term feedback prediction in greater detail for the first time in their article. I suggest to do so much earlier in the paper in order to facilitate the readers’ ability to understand this important subcomponent of the model. The authors then state that “As mentioned (see “social influence on the subjective situation”), manipulating the situation may often be a valid pathway to influence feedback prediction.” This suggests that social influence on one component of the model might simultaneously affect another one. Can the authors elaborate on this more? When outlining social influence on the individual components of the model earlier in the article, the examples given often appear to be applicable to more than one subcomponent (e.g., influencing the target’s action selection also affects the situation).

 The authors outline the studies by Melnyk et al. (2013) to show the influence of social norms onto action selection. Are they not also affecting action itself as well as participants’ goal states?

 The subsequently described studies could be better connected to the feedback loop model. The studies are also analysed regarding their limitations. At times, it is hard to see why these limitations are mentioned in the context of the authors’ model. The description of the study by Zhao and Pechmann (2007) is a good example for how to better connect the presented research with the theoretical model (especially the top paragraph of page 13).

 The authors state on page 13 that “This study is a good case of targeting action selection rather than goal structures”. A sentence like this at the beginning of each outlined study would help to understand why the authors are referring to these particular studies in light of their model.

 A possible way to better structure the description of the studies is to first outline what components of the model the studies target. After that, limitations can be described.

 On page 14, the authors state that “… it may be interesting to examine whether the discrepancy between the reference or goal state and the current state or situation can predict reactance. Potentially, a discrepancy that seems too large for the individual may promote reactance”. Assuming that the goal is the target's goal, wouldn't an enlarged discrepancy rather reduce reactance since the target would not see a point in pursuing the goal anyway?

 Also on page 14, the authors write “However, the affected stage largely depends on the intentions of the influencer, as the same influence tactic might be interpreted differently within another stage of the feedback loop”. I have troubles understanding this sentence. Please clarify

 As their very last sentence, the authors state that “understanding how to influence only one particular aspect while excluding the influence of another aspect may be crucial for a more detailed perspective on social influence.” I think this point represents a big argument for the model that should be emphasized more (also in the description of the described studies since it justifies and explains the authors’ effort to point out limitations and weaknesses of the mentioned studies).

 This brings me to the justification for introducing a feedback loop model in order to investigate social influence. In the introduction of the article, the authors describe past research on social influence. It appears that social influence has already been well studied and the question emerges what the proposed feedback loop model adds to this. The authors occasionally touch upon this question as on page 5 where they state that “While previous research has identified different strategies of social influence, our approach exceeds existing approaches by categorizing these strategies according to the five entrance gates to the internal feedback loop. Thereby, we show how our feedback framework can be used to distinguish and understand the different forms of social influence”. This justification should be mentioned earlier. In general, I would encourage the authors to make clearer why the feedback loop model is necessary and what advantages it entails (e.g., precise description of where and how different kinds of social influence affect a target).

 Lastly, I suggest some minor alterations and modifications for the nomenclature used in the article:

- The authors refer to different feedback loop concepts throughout the article. Among them is the feedback loop system (page 1), the cybernetic feedback model (page3 ), and the cybernetic feedback loop (page 10). An example for how this can lead to ambiguity can be found on page 4: “Feedback loops consist of several stages or elements, including the reference value, comparator, input, and output. In adapting the cybernetic feedback model to social influence…”. Here, it is hard to comprehend whether the authors refer to different concepts or whether they use the different terms interchangeably.
- On page 3, the authors write: “We aim to establish a theoretical connection between cybernetic control models and other feedback regulation models, such as reinforcement learning”. Reading this, I felt overwhelmed by the many terms and concepts introduced at ones. It might help to outline the term ‘cybernetic’ in greater detail. Furthermore, without having been introduced to the model, a reader can struggle to understanding where and how reinforcement learning comes into play here.
- I suggest to refrain from using the term ‘study’ (page 7 under “Social influence on action selection”) when referring to the theoretical model outlined in the present article. It suggests that data will be presented.

Is the topic of the opinion article discussed accurately in the context of the current literature?

Yes

Are arguments sufficiently supported by evidence from the published literature?

Yes

Are all factual statements correct and adequately supported by citations?

Partly

Are the conclusions drawn balanced and justified on the basis of the presented arguments?

Yes

Reviewer Expertise:

Social psychology, motivation, affect, approach-/avoidance-motivation, anger, powerlessness, threats

I confirm that I have read this submission and believe that I have an appropriate level of expertise to confirm that it is of an acceptable scientific standard, however I have significant reservations, as outlined above.

## References

[1] : To Have Control Over or to Be Free From Others? The Desire for Power Reflects a Need for Autonomy. *Personality and Social Psychology Bulletin* .2016;42(4) : 10.1177/0146167216634064 498-512 10.1177/0146167216634064 26984014

[2] : Striving for personal power as a basis for social power dynamics. *European Journal of Social Psychology* .2006;36(4) : 10.1002/ejsp.351 537-556 10.1002/ejsp.351

# Response

**Published:** 2023-12-21

**Authors:** Martin Weiß

**Affiliations:** University Hospital Würzburg, Germany

Dear Dr. Willardt,

 thank you for this thoughtful review. We have revised the paper based on the suggestions. See bellow, for our point-by-point response. As our manuscript is already quite long, we have tried to keep the revisions based on your comments as concise and precise as possible.

**The sentence “Different approaches have shown that social influence is related to emotional processes, visual attention, and self regulation” (page 3) feels like the justification for connecting a feedback loop model with social influence. If that is indeed the case, this should be stated more explicitly.**

*Response* *: *We agree and extended the sentence accordingly.

 “Different approaches have shown that social influence is related to emotional processes ( Fischer *et al.*, 2003), visual attention ( Frick *et al.*, 2018), and self-regulation ( vanDellen & Hoyle, 2010), thereby providing a connection between social influence and feedback loop models.”

**The term feedback control on page 3 (“In social interactions, actions elicit responses from others, leading to the possibility of feedback control…”) should be explained in greater detail.**

*Response* *: *We added the following explanation.

 “Feedback control in social interactions refers to the repeated process by which a person’s activities elicit reactions, generating a dynamic loop that determines subsequent behavior. This system requires constant adjustments depending on feedback and determines the continuing dynamics of interpersonal interactions.”

**On page 4 under the heading “Social influence”, a sentence is started with the word ‘nonetheless’, even though the preceding sentence does not entail a contradiction to the subsequent one.**

*Response* *: *We deleted “Nonetheless” from this sentence.

**It might be interesting for the discussion of social influence on page 4 that there is some power research indicating that power actually consists of two subcomponents, namely personal control and influence over others (e.g., Lammers et al, 2016; Van Dijke & Poppe, 2006).**

****

*Response:* We have refrained from a detailed discussion as we have already shortened the power paragraph to avoid distracting the reader from the main topic of this opinion piece. Nevertheless, we have added the aspect mentioned by the reviewer.

 “Like influence, social power has often been defined as the ability to control or influence another’s thoughts, behavior, or feelings in a meaningful way ( Fiske, 1993; Thibaut & Kelley, 1959; Vescio *et al.*, 2003). In this line, more recent research on social power indicates that power might consist of two subcomponents, namely personal control and influence over others (e.g., Lammers et al, 2016; Van Dijke & Poppe, 2006).”

**When describing the individual components of the feedback loop model, it would add clarity to specifically state whose goals are meant with the term “goal state”. Those of the target of those of the influencer? The same goes for the feedback prediction component.**

*Response:* Thank you. Since we are elaborating on how an influencer can intervene in the feedback loop of the target, it is always about the target. We have specified this in the respective sections.

**Is the comparator/reference component the plus/minus sign in the graphical depiction of the model? Please clarify.**

*Response:* Exactly, we have displayed the comparator with plus/minus signs, as it can lead to two results. Either there is a discrepancy exists or not. Discrepancies can be either positive or negative. We have clarified this in the figure caption:

 “The ± sign reflects the "comparator" and indicates that a possible discrepancy can be either positive or negative.” 

**On page 4 the authors introduce the struggle for a TV to illustrate the model. The goal in this case is described as “the division of a shared television”. But this seems to represent a communal goal rather than the individual goals of the two roommates. Maybe it would be better to avoid the term “goal” in this situation and only use it to refer to the target’s goals in the examples that follows.**

**In the subsequent sentence, the goal state is described as the target buying the television. The use of goal and goal state and the lack of a clear attribution of these terms to either one of the two roommates is very confusing.**

*Response: * We have grouped both of the reviewer's points together as they relate to the same paragraph. In this passage, we admit that the distinction between goal and goal state was confusing, as we sometimes referred to the TV as the goal and sometimes to a desire of the target (e.g., keeping the TV). We now always use goal for the TV and goal state for the target’s internal state. Thus, we changed the paragraph in the revised version as follows:

 “Imagine two people—an *influencer* and a *target—*arguing about the division of a currently shared television *.* Let us consider a situation where the *influencer* wants to buy a brand-new television for him- or herself. The *influencer* may try to increase the value of the currently shared object and negotiate a deal to “sell” the shared television to the *target* to receive some money, by manipulating the *target*’s **goal state** (a), that is ‘owning the shared TV’ in this example. In this situation, the *influencer* persuades the *target* to understand that the *goal* (i.e., the shared television) is a worthwhile undertaking with advantages over other goal-like items. Alternatively, the *influencer* may want to keep the shared television and try to devalue it (the goal) to get the *target* to buy a new one. In this situation, the *influencer* might persuade *the target* that the *goal* is not worthwhile. Both strategies directly manipulate the *target*’s internal goal state or reference; in these examples, the *influencer* tries to amplify or attenuate the attractiveness of the *goal* for the *target* by either increasing or decreasing the value attributed to the *goal *and thus changing the desirability of the goal state. Hence, the amplification, attenuation, implementation, and elimination of a goal state may be relevant principal target strategies.”

**See also the sentence “In this situation, the influencer might persuade the target that the goal is not worthwhile” where it is unclear what goal the authors refer to.**

*Response:* In our example, the goal is always the television. To make our example easier to understand, we have included the specific terms in brackets in the first paragraph in which we introduce the example.

**On page 5, the heading reads “Social influence towards the goal state”. This is somewhat irritating since social influence towards the goal state was already touched upon in the example of the two roommates (the same applies to the subsequent headings).**

*Response:* We have used the paragraph about the example with the two roommates and the TV to give a naturalistic overall impression of the proposed framework. The following headings serve to discuss the entrance gates in more detail with empirical findings. In our view, this is made clear by the last paragraph preceding the sections on the individual aspects:

 “To illustrate our theoretical views and contribution to the literature, we provide specific examples of negotiation, social norms and sanctions, compliance, advertisement, and nudging in the following subsections. While previous research has identified different strategies of social influence ( Bruins, 1999; Higgins *et al.*, 2003), our approach exceeds existing approaches by categorizing these strategies according to the five entrance gates to the internal feedback loop. Thereby, we show how our feedback framework can be used to distinguish and understand the different forms of social influence.”

**Here, the authors should also explain why a differentiation between persuasion and manipulation is necessary.**

*Response:* We provided the following explanation.

 “A differentiation between persuasion and manipulation is necessary since persuaders usually have transparent intentions and are open about their goals, whereas manipulators may hide their true intentions, using tactics that intentionally mislead or deceive others.”

**The sentence “Influence through persuasion can affect the goal state even before the target takes any action. Persuasion is the most appropriate term for what we consider influence toward the goal state” is hard to comprehend: At this point it is yet unclear what the difference between influence and persuasion is. Is persuasion a sub-facet of influence, namely influence towards the goal state? Please explain.**

*Response:* When we talk about influence directed toward achieving a particular goal or outcome, especially through communication and reasoning, the term "persuasion" is the most fitting descriptor. It's not that persuasion is a sub-facet of influence; rather, it's a specific type of influence with a focus on communication and achieving a defined goal state. However, we understand that our initial formulation needs to be improved.

 “For influence directed toward achieving a particular goal, especially through communication and reasoning, the term "persuasion" is the most fitting descriptor. We do not aim at conceptualizing persuasion is a sub-facet of influence; rather, we use persuasion as a specific type of influence with a focus on communication and achieving a defined goal state.”

**The terms ‘message elaboration’ and ‘peripheral route’ should also be outlined in greater detail**

*Response:* We added explanations for both aspects.

 “Message elaboration refers to the extent to which individuals actively process information contained in a message. Thus, it involves the cognitive effort and depth of thinking invested in understanding and evaluating a message. In contrast, when a person lacks the ability or motivation to process a message in-depth, they may rely on the peripheral route, which means that peripheral cues or superficial aspects of the message rather than engaging in elaborate cognitive processing. These peripheral cues could be the influencer’s attractiveness or level of knowledge, which could lead to increased credibility.”

**The authors refer to Sherif’s experiment (p. 7). Outlining the mentioned goal the manipulator is thought to influence (I assume it is the social goal to give group-conform responses) would help to better imbed this research.**

*Response: * We have gratefully integrated the useful addition provided by the reviewer.

 “A manipulator can influence goals to trigger conformity (e.g., the social goal to give group-conform responses), as shown by an experiment in which people estimated the movement of a dot of light ( Sherif, 1936).”

**Under the heading “Social influence on the subjective situation” on page 7, the authors refer to social comparison. The connection of this concept to the example given and to social influence on the situation remains somewhat unclear.**

*Response:* In line with the reviewer’s comment, we emphasized the connection between social comparison and influence on the situation. 

 “Social comparison is an adjacent concept that alters the perception of a situation instead of the facts. Social comparison involves comparing oneself to others, which can influence how individuals perceive their own circumstances. Hence, the influencer's actions may lead the target making upward and downward social comparisons. Upward comparisons aim to increase achievement, while downward comparisons aim to increase subjective well-being ( Diel et al., 2021; Wheeler, 1966; Wills, 1981). Accordingly, the target may perceive the situation differently when making either of these two kinds of comparisons.”

**On page 8, the authors write “In the context of the shared TV example, imagine that the influencer wants to donate the TV to charity, but the target wants to keep it.” In that case the goal state of the target would be to keep the TV. I suggest to emphasize the connection between the used examples (such as this one) and the components of the proposed model more (in this case: target’s goal à keep TV).**

*Response:* Thank you for pointing this out. We rewrote the section to clarify this in the mentioned paragraph.

**“**In contrast, to make some action options less likely, the opposite would be promising, namely a reduction in the expected agency and sense of control. In the context of the shared TV example, imagine that the *influencer* wants to donate the TV to charity, but the *target’s *goal state would be to keep it. If the *influencer* posts about giving it to charity on social media, knowing the *target* will see it, it could lead to the *target* withdrawing their own action plan to get the TV since they might feel unable to oppose such a socially desirable action option. In this case, the *influencer* would have promoted an alternative course of action by raising doubts in the target about their own potential action. The *action selection* in the *target* may thus result in withdrawing from keeping the TV and the decision to let it go. This influence strategy is based on the *target*’s re-evaluation processes and could be successful for the *influencer.*”

**Also on page 8, the authors write “If the target perceives the influencer as highly reputable and trustworthy, they may be more likely to obey or comply with the influencer’s advice. This is true, even if the advice does not necessarily align with the target’s intuitive approach to achieving their goals.” This empirical claim should be backed up by a reference.**

*Response* *: *Agreed. We added the following references.

 Haslam, S. A., Reicher, S. D., & Birney, M. E. (2014). Nothing by mere authority: Evidence that in an experimental analogue of the Milgram paradigm participants are motivated not by orders but by appeals to science. *Journal of Social Issues*, *70*(3), 473-488.

 Hollander, M. M., & Turowetz, J. (2017). Normalizing trust: Participants’ immediately post‐hoc explanations of behaviour in M ilgram's ‘obedience’experiments. *British Journal of Social Psychology*, *56*(4), 655-674.

**Under the heading “Social influence on behavior while acting”, the authors write “The social influence induced by social norms can alter the internal feedback loop regarding action selection.” This implies that the authors refer to the subcomponent action selection rather than the subcomponent action here (see also the subsequent sentence). It would help the clarity of the paper to precisely state which subcomponents the authors target.**

*Response* *: *We specified the potential influence on the action itself more clearly in this paragraph.

 “The social influence induced by social norms can alter the internal feedback loop regarding action selection but also action itself. Specifically, exposure to peer groups’ behavior can prime and make a selected action more salient ( Do et al., 2022) or the mere adaption of an action more likely ( Albert et al., 2013). **”**

**Under the heading “Social influence on feedback prediction”, the authors outline the term feedback prediction in greater detail for the first time in their article. I suggest to do so much earlier in the paper in order to facilitate the readers’ ability to understand this important subcomponent of the model. The authors then state that “As mentioned (see “social influence on the subjective situation”), manipulating the situation may often be a valid pathway to influence feedback prediction.” This suggests that social influence on one component of the model might simultaneously affect another one. Can the authors elaborate on this more? When outlining social influence on the individual components of the model earlier in the article, the examples given often appear to be applicable to more than one subcomponent (e.g., influencing the target’s action selection also affects the situation).**

*Response* *: *We agree with the reviewer that the influence on one component may not only affect this component, but also other components. We have included a brief explanation on this topic before presenting all the individual components in detail.

 “Prior to an in-depth exploration of each component within our proposed framework in detail, we want to clarify that our discussion emphasizes the predominant influence on individual components. This focus does not mean that influencing one component has no effect on other components. For instance, influencing the *target*'s action selection could also affect the goal state or situation. This interplay among components underscores the complexity of social influence, suggesting that interventions on one component may cascade into multifaceted consequences. However, our primary goal is to delve into the fundamental elements of influencing another person’ internal feedback loop. Developing an understanding of how to influence only one particular aspect while excluding the influence of another aspect can be crucial for a more detailed perspective on social influence.”

 We also extended the section on feedback prediction in the introduction:

 “Influencing the target’s feedback prediction (d) mechanism might motivate or discourage them by enhancing or lessening their expectations of achieving the goal. For example, the influencer could say, “I will make it very difficult for you to get this TV!” or “Why bother buying a new TV when you can have this one easily?”. Hence, the action might be encouraged or hindered by manipulating expectations; in particular, lowering the target’s expectations of success might result in frustration and disappointment, leading them to discontinue potential further efforts. Accordingly, feedback prediction mainly refers to the likelihood of reaching the desired goal state when selecting a certain action.”

**The authors outline the studies by Melnyk et al. (2013) to show the influence of social norms onto action selection. Are they not also affecting action itself as well as participants’ goal states?**

*Response* *: S*ince previous studies do not build on our framework, it is not trivial to select exemplary research to illustrate the application of the model. We agree that some of the studies concern not only the part for which we selected them, but also other parts of the model. In the previous version of the manuscript, we already informed the reader about these overlaps, but have now added an extension.

 “There may be overlapping aspects between – but also within – the studies regarding the different entrance gates to manipulate the *target*’s feedback loop, which we will present in the following subsections.“

**The subsequently described studies could be better connected to the feedback loop model. The studies are also analysed regarding their limitations. At times, it is hard to see why these limitations are mentioned in the context of the authors’ model. The description of the study by Zhao and Pechmann (2007) is a good example for how to better connect the presented research with the theoretical model (especially the top paragraph of page 13).**

**The authors state on page 13 that “This study is a good case of targeting action selection rather than goal structures”. A sentence like this at the beginning of each outlined study would help to understand why the authors are referring to these particular studies in light of their model.**

**A possible way to better structure the description of the studies is to first outline what components of the model the studies target. After that, limitations can be described.**

*Response* *: *Thank you. In the manuscript, we already explained our systematic approach in more detail before discussing the concrete examples: “Before starting with the research examples, we will briefly describe the systematic approach used to analyze studies in this domain. First, we identified the relevant goals in the experiment ( *e.g.,* social goals, personal goals) and whether their value was manipulated or an additional goal was included. For instance, introducing a social comparison may add social goals to an otherwise private context. Second, we analyzed the influenced situational characteristics. Third, we examined whether action selection was manipulated—whether a specific behavioral choice was promoted. Fourth, we examined action opportunities or direct effects on action execution. Fifth, we examined the effects of social influence on feedback prediction and goal state versus situation discrepancy, for example, by manipulating a *target’s* subjective goal attainment probability.

 In the following subsections, we explain the findings, apply our model, and derive additional experimental manipulations based on our framework.”

 To address the reviewer’s point without losing our systematic structure, we have added/revised the introductory sentences of each example to make it clearer which component(s) of our model are covered in more detail.

**On page 14, the authors state that “… it may be interesting to examine whether the discrepancy between the reference or goal state and the current state or situation can predict reactance. Potentially, a discrepancy that seems too large for the individual may promote reactance”. Assuming that the goal is the target's goal, wouldn't an enlarged discrepancy rather reduce reactance since the target would not see a point in pursuing the goal anyway?**

*Response* *: *This is an interesting thought experiment. We admit that there could be a sweet spot where the discrepancy could become large enough that the goal pursuit could become obsolete, which would mean that the reactance would be reduced. We have extended the sentence to this point.

 “Alternatively, there may be a threshold at which the magnitude of the discrepancy between the goal state and the current state renders the pursuit of the goal obsolete, thus resulting in a decline in reactance.”

            

**Also on page 14, the authors write “However, the affected stage largely depends on the intentions of the influencer, as the same influence tactic might be interpreted differently within another stage of the feedback loop”. I have troubles understanding this sentence. Please clarify**

*Response* *: *We revised this sentence as follows:

 “Depending on the *influencer’s *intentions, one specific influence tactic might affect different stages or even more than one stage at the same time.”

**As their very last sentence, the authors state that “understanding how to influence only one particular aspect while excluding the influence of another aspect may be crucial for a more detailed perspective on social influence.” I think this point represents a big argument for the model that should be emphasized more (also in the description of the described studies since it justifies and explains the authors’ effort to point out limitations and weaknesses of the mentioned studies).**

*Response* *: *This is a valid and very helpful suggestion. We incorporated this aspect in the section we inserted before the in-depth discussion of the components of our framework.

 “Prior to an in-depth exploration of each component within our proposed framework in detail, we want to clarify that our discussion emphasizes the predominant influence on individual components. This focus does not mean that influencing one component has no effect on other components. For instance, influencing the *target*'s action selection could also affect the goal state or situation. This interplay among components underscores the complexity of social influence, suggesting that interventions on one component may cascade into multifaceted consequences. However, our primary goal is to delve into the fundamental elements of influencing another person’ internal feedback loop. Developing an understanding of how to influence only one particular aspect while excluding the influence of another aspect can be crucial for a more detailed perspective on social influence.”

**This brings me to the justification for introducing a feedback loop model in order to investigate social influence. In the introduction of the article, the authors describe past research on social influence. It appears that social influence has already been well studied and the question emerges what the proposed feedback loop model adds to this. The authors occasionally touch upon this question as on page 5 where they state that “While previous research has identified different strategies of social influence, our approach exceeds existing approaches by categorizing these strategies according to the five entrance gates to the internal feedback loop. Thereby, we show how our feedback framework can be used to distinguish and understand the different forms of social influence”. This justification should be mentioned earlier. In general, I would encourage the authors to make clearer why the feedback loop model is necessary and what advantages it entails (e.g., precise description of where and how different kinds of social influence affect a target).**

*Response* *: *In line with the reviewer’s suggestion, we shifted the motivation for our model to an earlier point in the manuscript (section “Introduction”) and refined the necessity of such a model.

 “We propose a framework that identifies beneficial and detrimental manipulation tactics in everyday life, contributing to a broader understanding of social influence.  While previous research has identified different strategies of social influence ( Bruins, 1999; Higgins et al., 2003), our approach exceeds existing approaches by categorizing these strategies according to distinct entrance gates to the internal feedback loop. Thereby, the proposed framework helps to develop a precise description of where and how different kinds of social influence affect another person’s internal feedback loop.”

**Lastly, I suggest some minor alterations and modifications for the nomenclature used in the article: **

- **The authors refer to different feedback loop concepts throughout the article. Among them is the feedback loop system (page 1), the cybernetic feedback model (page3 ), and the cybernetic feedback loop (page 10). An example for how this can lead to ambiguity can be found on page 4: “Feedback loops consist of several stages or elements, including the reference value, comparator, input, and output. In adapting the cybernetic feedback model to social influence…”. Here, it is hard to comprehend whether the authors refer to different concepts or whether they use the different terms interchangeably.**

*Response* *: *We understand the confusion and deleted the sentence on page 4 as it was also redundant (see first sentence in the section “Feedback loops and feedback control”). To avoid misunderstandings, we used the term cybernetic feedback model consistently.

**On page 3, the authors write: “We aim to establish a theoretical connection between cybernetic control models and other feedback regulation models, such as reinforcement learning”. Reading this, I felt overwhelmed by the many terms and concepts introduced at ones. It might help to outline the term ‘cybernetic’ in greater detail. Furthermore, without having been introduced to the model, a reader can struggle to understanding where and how reinforcement learning comes into play here.**

*Response* *: *We improved the order of our arguments as the reinforcement learning part was more like a future use case of a theoretical connection to our model. The revised paragraphs are as follows:

 “We build on cybernetic models of behavioral regulation, that is, models that are designed to understand or regulate dynamic processes in complex systems ( Leonard et al., 2021), focusing on the fundamental elements that comprise these models. Cybernetic models illustrate a closed system that regulates itself using a feedback loop. Social influence can target elements in cybernetic models, representing different forms of social influence.

 We propose a framework that identifies beneficial and detrimental manipulation tactics in everyday life, contributing to a broader understanding of social influence.  While previous research has identified different strategies of social influence ( Bruins, 1999; Higgins et al., 2003), our approach exceeds existing approaches by categorizing these strategies according to distinct entrance gates to the internal feedback loop. Thereby, the proposed framework helps to develop a precise description of where and how different kinds of social influence affect another person’s internal feedback loop.

 Our framework helps establishing a theoretical connection between cybernetic control models and other feedback regulation models, such as reinforcement learning ( i.e., learning the optimal behavior in an environment to obtain maximum reward through observations of how choices are influenced by past decisions and rewards; Sutton & Barto, 1998). By combining these models in the future, researchers might be able to identify interactions that enhance our understanding and prediction of social influence. For example, combining our proposed model with reinforcement learning can help determine which social influence mechanisms are advantageous and crucial in social interactions.”

**I suggest to refrain from using the term ‘study’ (page 7 under “Social influence on action selection”) when referring to the theoretical model outlined in the present article. It suggests that data will be presented.**

*Response* *: *Agreed. We replaced the term “study” by “article” or “opinion piece”.

# Reviewer report: Reviewer response for version 1

**DOI:** 10.5256/f1000research.146274.r187780

**Published:** 2023-08-07

**Authors:** Yanyan Qi

**Affiliations:** 1 School of Education, Zhengzhou University, Zhengzhou, Henan, China

In this article, Weiß et al. proposed a social influence model under a cybernetic control system. They hypothesized that social influence can occur by influencing the target’s situation, goal state, comparator, action selection, feedback predictor, or action. Then as model applications, they discussed some commonly-seen social influence tactics, e.g., social norms, and advertisement, under this framework. This paper is interesting and in general easy to follow. While before indexing, I still have some suggestions.

 Abstract

1. In the first sentence the authors said “This article aims to describe social influence by elucidating the cognitive, affective, and motivational processes that occur when an individual experiences an attempt at being influenced.” But when reading the full context, I am still confused about the meaning of “the cognitive, affective, and motivational processes”.
2. I suggest the authors write the abstract according to the manuscript, e.g., the author mentioned ‘ coercion targets the selection mechanism, and violence targets the action’, which had not been discussed in the full text. 

The full context

1. Since the paper is quite long, I suggest the authors give different heading levels, which will make it easier to understand the article structure. 
2. In “social influence on behaviour while acting” section, the authors first mentioned “descriptive norms” without the definition, while in “Examples of model applications” section, the authors mentioned it again and gave a definition. Please re-organize this part. 
3. Some newly published literature could be added to the manuscript.
4. Just a suggestion, since the author frequently mentioned “framing”, and “self-regulatory focus”, I am thinking maybe you can add an “influencing factors” section, which can contain not only the “framing” and “self-regulatory focus” from the target’s side, but also the characteristics of the influencer, e.g., as you mentioned, reputation and trustworthiness.

Is the topic of the opinion article discussed accurately in the context of the current literature?

Yes

Are arguments sufficiently supported by evidence from the published literature?

Yes

Are all factual statements correct and adequately supported by citations?

Yes

Are the conclusions drawn balanced and justified on the basis of the presented arguments?

Partly

Reviewer Expertise:

Social psychology, social behavior, and social emotions

I confirm that I have read this submission and believe that I have an appropriate level of expertise to confirm that it is of an acceptable scientific standard, however I have significant reservations, as outlined above.

# Response

**Published:** 2023-12-21

**Authors:** Martin Weiß

**Affiliations:** University Hospital Würzburg, Germany

Dear Dr. Qi,

 we appreciate the thoughtful review and have addressed the feedback. These changes are noted below in a point-by-point response.

**Abstract**

**In the first sentence the authors said “This article aims to describe social influence by elucidating the cognitive, affective, and motivational processes that occur when an individual experiences an attempt at being influenced.” But when reading the full context, I am still confused about the meaning of “the cognitive, affective, and motivational processes”.**

*Response* *: *We agree that this sentence is too vague and not well explained in the abstract. Therefore, we have reworded the beginning of the abstract as follows:

 “This article aims to unravel the dynamics of social influence by examining the processes that occur when one person is the target of another's influence.”

**I suggest the authors write the abstract according to the manuscript, e.g., the author mentioned ‘ coercion targets the selection mechanism, and violence targets the action’, which had not been discussed in the full text.**

*Response* *: *We changed “coercion” to “obedience” as an example for the selection mechanism as we discussed obedience in more detail in the corresponding section. For action, however, we stuck to “violence”, as this was one of our examples (“violent or deceptive action redirections”).

**The full context**

**Since the paper is quite long, I suggest the authors give different heading levels, which will make it easier to understand the article structure.**

*Response* *:* The heading levels were set to one level by the journal during the editing process - we unfortunately cannot change anything in this regard.

****

**In “social influence on behaviour while acting” section, the authors first mentioned “descriptive norms” without the definition, while in “Examples of model applications” section, the authors mentioned it again and gave a definition.** **Please re-organize this part.**

*Response* *: *Thank you. Agreed. We moved the definition to the first mentioned section “Social influence on behavior while acting”. The first sentence of the second paragraph in this section now reads:

 “However, most social influence research concerns social norms and subtle psychological processes ( Cialdini & Goldstein, 2004). Thereby, descriptive norms refer to the behavior of relevant others ( *e.g.,* peers, family, society) and provide a socially determined standard for effective behavior ( *i.e.,* what individuals typically do), while injunctive norms refer to appeals about what other members of society approve or disapprove of.”

****

**Some newly published literature could be added to the manuscript.**

*Response* *: *We have included the following (more recent) papers at appropriate positions in the manuscript.

 Caspar, E. A., Beyer, F., Cleeremans, A., & Haggard, P. (2021). The obedient mind and the volitional brain: A neural basis for preserved sense of agency and sense of responsibility under coercion. *PloS one*, *16*(10), e0258884.

 Diel, K., Grelle, S., & Hofmann, W. (2021). A motivational framework of social comparison. *Journal of Personality and Social Psychology, 120*(6), 1415–1430.

 Gass, R. H., & Seiter, J. S. (2022). *Persuasion: Social influence and compliance gaining*. Routledge.

 Haslam, S. A., Reicher, S. D., & Birney, M. E. (2014). Nothing by mere authority: Evidence that in an experimental analogue of the Milgram paradigm participants are motivated not by orders but by appeals to science. *Journal of Social Issues*, *70*(3), 473-488.

 Hollander, M. M., & Turowetz, J. (2017). Normalizing trust: Participants’ immediately post‐hoc explanations of behaviour in M ilgram's ‘obedience’experiments. *British Journal of Social Psychology*, *56*(4), 655-674.

 Legros, S., & Cislaghi, B. (2020). Mapping the social-norms literature: An overview of reviews. *Perspectives on Psychological Science*, *15*(1), 62-80.

 Ren, Q., Gentsch, A., Kaiser, J., & Schütz-Bosbach, S. (2023). Ready to go: higher sense of agency enhances action readiness and reduces response inhibition. *Cognition*, *237*, 105456.

 Sosa, M., & Giocomo, L. M. (2021). Navigating for reward. *Nature Reviews Neuroscience*, *22*(8), 472-487.

 Spears, R. (2021). Social influence and group identity. *Annual Review of Psychology*, 72, 367-390.

 Wolske, K. S., Gillingham, K. T., & Schultz, P. W. (2020). Peer influence on household energy behaviours. *Nature Energy*, *5*(3), 202-212.

**Just a suggestion, since the author frequently mentioned “framing”, and “self-regulatory focus”, I am thinking maybe you can add an “influencing factors” section, which can contain not only the “framing” and “self-regulatory focus” from the target’s side, but also the characteristics of the influencer, e.g., as you mentioned, reputation and trustworthiness.**

*Response* *: *We appreciate the suggestion by the reviewer. Since this opinion piece is focused on how and where the influencer can enter the internal feedback loop system of the target, we have deliberately decided not to address individual differences of the influencer, because we believe that the focus of the paper would otherwise remain less clear and it would be too much for the manuscript in terms of its scope. We have therefore refrained from further elaborating on “influencing factors” in relation to the influencer him/herself although this is a highly interesting topic.
